# A Universal Bio-Hybrid Nanoparticle Backpack Platform Endows Stem Cells with Microenvironmental Resilience and Sustained Paracrine Signaling

**DOI:** 10.1007/s40820-026-02308-3

**Published:** 2026-07-20

**Authors:** Yuqing Chen, Ying Yang, Shuo Yang, Xingyi Shu, Zhiyong Liu, Jian Song, Ya-Xuan Zhu, Han Lin, Ruili Wei, Jianlin Shi

**Affiliations:** 1 Department of Ophthalmology, Shanghai Changzheng Hospital, Second Affiliated Hospital of Naval Medical University, Shanghai, 200003 People’s Republic of China; 2 Department of Ophthalmology, Wuxi No. 2 People’s Hospital, Wuxi, 214000 People’s Republic of China; 3 Shanghai Tenth People’s Hospital, Shanghai Frontiers Science Center of Nanocatalytic Medicine, School of Medicine, Tongji University, Shanghai, 200072 People’s Republic of China; 4 State Key Laboratory of High Performance Ceramics, Shanghai Institute of Ceramics Chinese Academy of Sciences, Shanghai, 200050 People’s Republic of China

**Keywords:** Bio-hybrid nanoplatform, Engineered mesenchymal stem cells, Mesoporous silica backpack, Sustained paracrine signaling, Microenvironmental resilience

## Abstract

The therapeutic potential of mesenchymal stem cells (MSCs) in regenerative medicine is frequently thwarted by hostile post-injury microenvironments characterized by oxidative stress, inflammation, and rapid clearance, which compromise cell survival and paracrine efficacy. Herein, we establish a universal bio-hybrid “stem cell backpack” platform designed to fundamentally overcome these bottlenecks through precise surface interface engineering. By tethering drug-loaded mesoporous silica nanoparticles onto the MSCs membrane via mild, biocompatible click chemistry, we create a programmable nanotherapeutic depot that endows host cells with dual, synergistic capabilities: (1) robust scavenging of reactive oxygen species (ROS) to ensure cellular resilience and (2) sustained, localized release of regenerative factors to amplify paracrine signaling. Using corneal chemical injury as a rigorous proof-of-concept model, this platform demonstrated unprecedented regenerative potency. The engineered cells markedly outperformed conventional therapies by simultaneously suppressing inflammatory cascades, preventing fibrosis and neovascularization, and orchestrating multi-lineage regeneration, including epithelial restoration, nerve reinnervation, and limbal stem cell reactivation. Quantitatively, this approach achieved a 63.6% enhancement in transparency restoration and a 76.9% greater reduction in tissue defects compared to cell monotherapy. Crucially, the modular design of this backpack system allows for the interchangeable loading of diverse therapeutics, extending its applicability beyond ophthalmology to other tissues facing similar microenvironmental challenges. This work presents a transformative paradigm in nanomedicine, shifting the focus from passive cell delivery to active microenvironmental modulation, thereby offering a versatile and scalable strategy for next-generation regenerative therapies across diverse clinical indications.

**Supplementary Information:**

The online version contains supplementary material available at 10.1007/s40820-026-02308-3.

## Highlights

- A universal bio-hybrid stem cell backpack platform shifts regenerative nanomedicine from passive cell delivery to active microenvironmental modulation.
- The modular nano-depot endows stem cells with dual synergistic capabilities: robust reactive oxygen species scavenging for cellular resilience and localized factor release for amplified paracrine signaling.
- Demonstrated in a rigorous corneal injury model, the system promotes multi-lineage regeneration and tissue repair, potentially via NF-κB/MAPK pathway modulation, offering a scalable strategy for diverse clinical applications.

**Supplementary Information:**

The online version contains supplementary material available at 10.1007/s40820-026-02308-3.

## Graphical abstract

**Figure:**
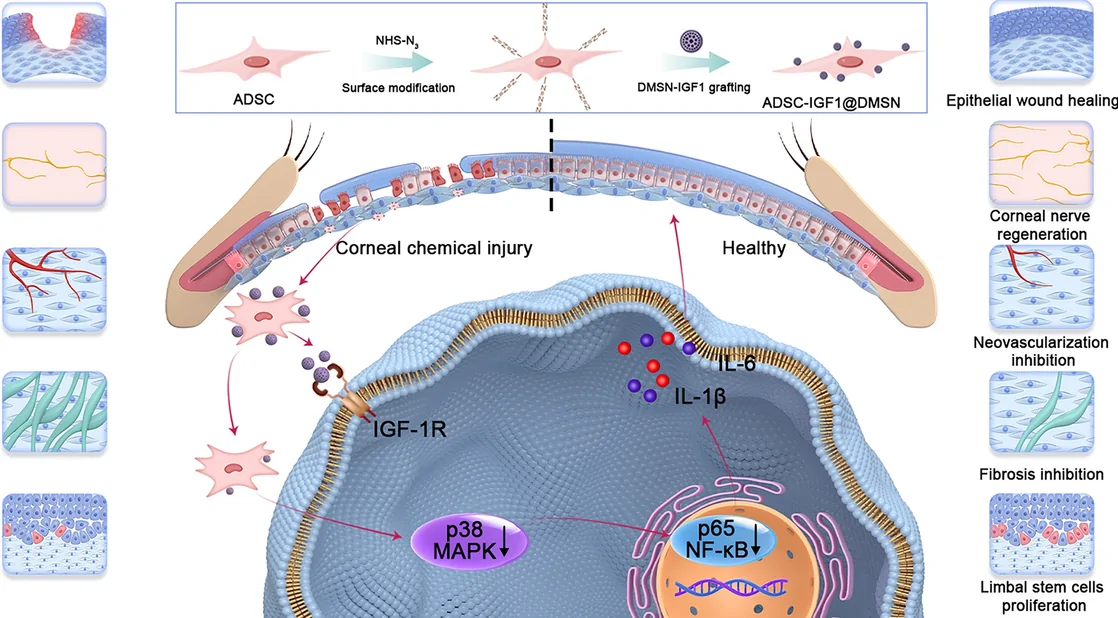

## Introduction

Mesenchymal stem cells (MSCs)-based therapy has emerged as a promising strategy for regenerative medicine due to its immunomodulatory capacity, trophic support, and ability to respond to injury-associated cues [1–3]. However, the therapeutic efficacy of MSCs is often substantially thwarted after transplantation, not simply by insufficient delivery, but by the hostile post-injury microenvironment itself [4, 5]. Oxidative stress, excessive inflammatory signaling, and rapid clearance collectively impair stem cell survival, persistence, and paracrine activity, limiting the reparative capacity of cell-based therapies [6, 7]. Therefore, a major challenge in regenerative medicine is to develop strategies that not only deliver MSCs to injured tissues, but also preserve their functionality and amplify their instructive signaling within damaged microenvironments [8].

To improve stem cell therapies, researchers have explored various biomaterial-assisted approaches, including hydrogels, scaffolds, and nanodevices designed to enhance cell retention or drug delivery [9–12]. Although these strategies have yielded significant progress, they still face notable limitations. Soluble therapeutic factors are frequently limited by poor local bioavailability and rapid clearance, whereas nanomaterials alone often lack the adaptive biological communication, tissue responsiveness, and injury homing potential of living cells [7, 13]. Conversely, unmodified MSCs can migrate toward inflamed tissues and provide endogenous paracrine support, but they remain highly susceptible to oxidative and inflammatory damage following administration [14]. Based on these considerations, an effective solution should integrate the biological adaptability of stem cells with the programmability of nanomaterials at the cell-microenvironment interface [15–17]. In principle, this bio-hybrid design can enhance the resilience of therapeutic cells in hostile microenvironments while enabling sustained local delivery of regenerative signals.

Corneal chemical injury (CCI) represents a notably rigorous proof-of-concept model for assessing such a strategy [18–20]. As a severe form of ocular surface damage, CCI induces acute oxidative stress, excessive inflammation, epithelial and stromal disruption, neovascularization, fibrosis, and profound impairment of corneal homeostasis [21, 22]. Consequently, effective therapy requires more than rapid wound closure. Full functional recovery necessitates coordinated regeneration of multiple tissue components, including epithelial restoration, barrier reconstruction, suppression of inflammation and fibrosis, corneal transparency recovery, nerve reinnervation, and activation of limbal stem cells (LSCs) [23, 24]. Adipose tissue-derived mesenchymal stem cells (ADSCs), owing to their accessibility, low immunogenicity, and reparative potential, have shown promise in ocular surface repair [25–27]. Nevertheless, ADSC monotherapy often fails to achieve complete restoration under severe injury conditions, as the hostile microenvironment limits cell survival and compromises paracrine signaling [28, 29]. Thus, CCI represents not only a clinically relevant injury model, but also a rigorous model for evaluating whether an engineered stem cell system can orchestrate multidimensional regeneration under hostile microenvironmental conditions.

**Fig. 1:**
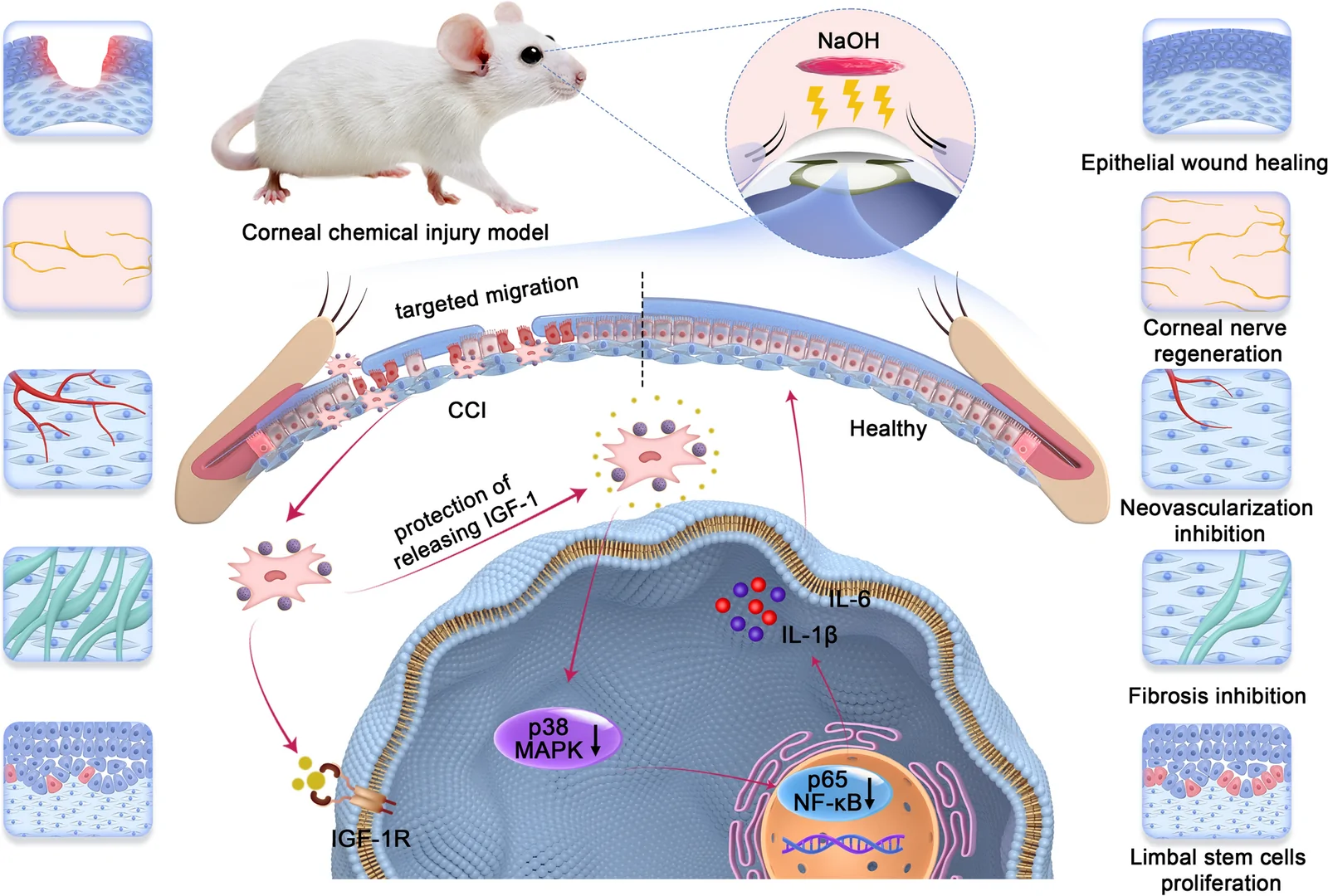
Schematic diagram of the universal bio-hybrid stem cell backpack platform and its therapeutic mechanism in corneal injury repair

Herein, we present a universal bio-hybrid stem cell backpack platform established through precise cell surface nanoengineering to actively program therapeutic cells against hostile microenvironments. In this design, dendritic mesoporous silica nanoparticles (DMSNs) act as modular nanoscale depots for cargo loading and sustained release [30–32], while ADSCs serve as NF-κB/MAPK responsive cellular chassis and biological effectors [33]. IGF-1 is anchored to the ADSC membrane through mild bioorthogonal click chemistry, thereby enhancing cellular resilience to oxidative stress while sustaining localized factor release and strengthening paracrine signaling [34, 35]. Using CCI as a rigorous proof-of-concept model, we demonstrate that this engineered system promotes multidimensional corneal regeneration. Specifically, it enhances epithelial repair, restores transparency, supports corneal nerve reinnervation, and activates limbal stem cells. Concurrently, it suppresses inflammation, fibrosis, neovascularization, and lymphangiogenesis. Mechanistically, these effects are linked to attenuation of NF-κB/MAPK-driven inflammatory cascades and establishment of a pro-regenerative niche. More broadly, the modular and cargo-interchangeable nature of this platform positions it as a versatile strategy that shifts regenerative cell therapy from passive delivery to active microenvironmental modulation (Fig. 1).

## Experimental Section

See the Supporting Information for the experimental details.

## Results and Discussion

### Construction and Interfacial Engineering of a Modular Stem Cell Backpack Platform

**Fig. 2:**
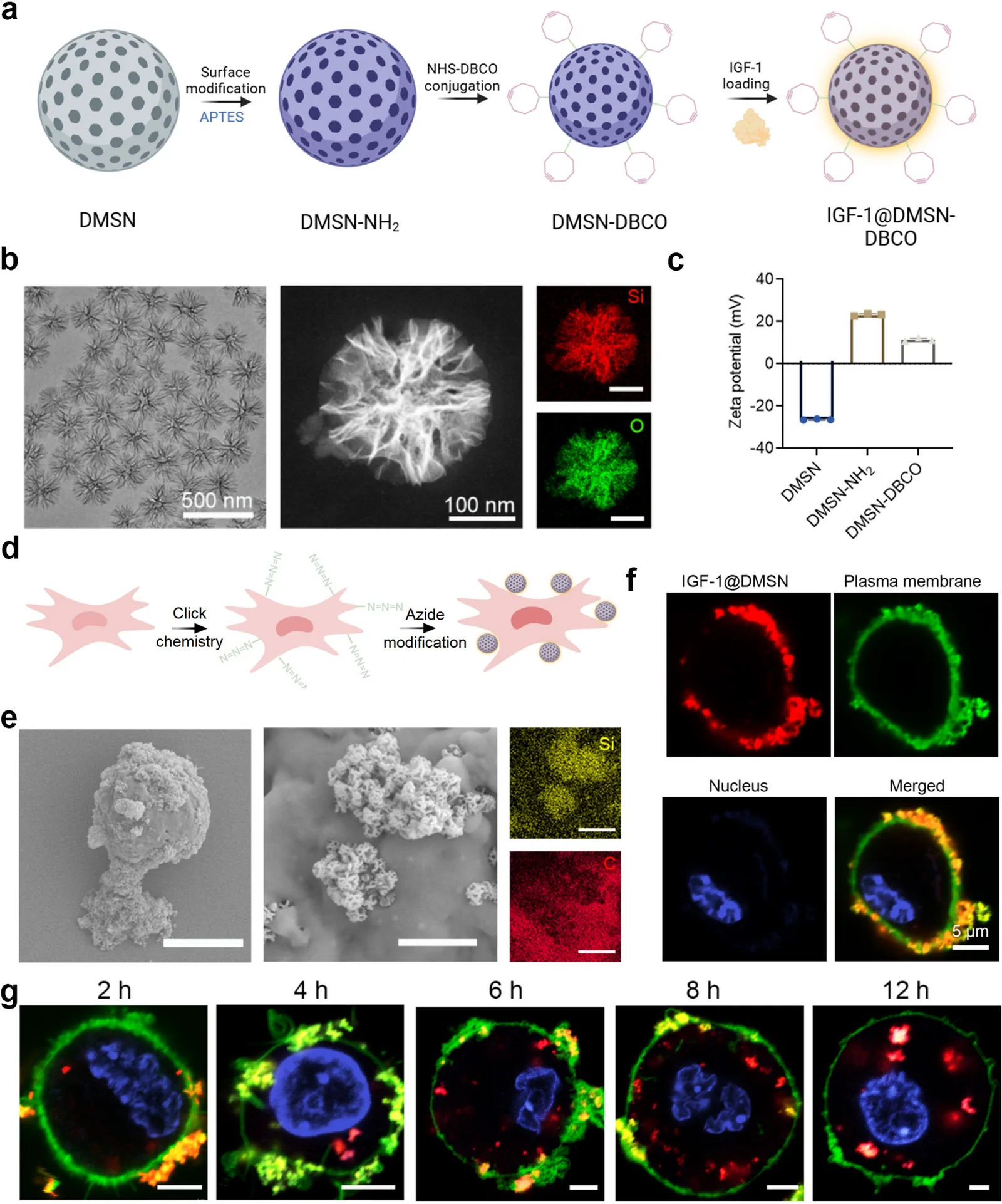
Fabrication and characterization of the engineered stem cell backpack platform.** a** Scheme illustrating the fabrication of the stem cell backpack (IGF1@DMSN-DBCO). **b** TEM image and elemental distribution of DMSN. **c** Zeta potentials of DMSN, DMSN-NH_2_, and DMSN-DBCO nanoparticles. **d** Schematic of cell surface conjugation of backpacks onto ADSCs. **e** SEM images of ADSC-IGF1@DMSN. **f** Confocal images of ADSCs conjugated with Cy5-labeled backpacks. (Nucleus: Hoechst 33,342; Plasma membrane: Chol-PEG-FITC). **g** Time-dependent distribution of backpacks on ADSCs

To realize a universal bio-hybrid platform capable of protecting therapeutic cells while enabling sustained local signal delivery, we first built a modular stem cell surface “backpack” that enables sustained and localized IGF-1 delivery by combining a DBCO-functionalized dendritic mesoporous silica carrier via a click reaction under mild conditions (Fig. 2a). In this design, DMSN were synthesized in a one-pot process using CTAB/NaSal as the structure-directing system, TEOS as the silica precursor, and TEA as the catalyst. The as-prepared DMSNs serves as the main scaffold of the “backpack”, providing a porous channel network for IGF-1 loading and protection. DMSNs was then aminated with APTES, followed by conjugation with NHS-DBCO to yield DBCO-functionalized “backpacks” for subsequent cell surface click coupling. TEM revealed that the DMSNs were highly uniform, with a characteristic dendritic architecture and an average size of ~ 200 nm (Fig. 2b). The open pore network provides sufficient capacity for IGF-1 loading, and elemental mapping confirmed the silica-based framework (Fig. 2b). Consistent with surface modification, pristine DMSNs showed a negative zeta potential of approximately − 20 mV, which shifted to a positive value after surface amine functionalization by APTES, supporting successful introduction of amino groups. After DBCO grafting, the surface potential became slightly less positive, likely because a fraction of the amines was consumed during conjugation (Fig. 2c). The experimentally determined DBCO loading (0.065 µmol mg^−1^) corresponds to a conjugation efficiency of 84.4%. This high efficiency indicates a successful conjugation under the employed conditions (Figs. S1 and S2). ELISA measurements gave an IGF-1 encapsulation efficiency of 5.8%. To functionalize the cell surface with the backpacks, we first introduced azide groups onto ADSCs using NHS-PEG-N_3_ and then incubated the azide-presenting cells with IGF1@DMSN-DBCO to obtain IGF1@DMSN-decorated ADSCs via strain-promoted azide-alkyne cycloaddition (Fig. 2d). SEM images showed that the backpacks were readily anchored on the cell surface, and confocal microscopy further indicated good co-localization with the plasma membrane (Fig. 2e, f). Additionally, we adapted flow cytometry to optimize and quantify surface grafting levels (Fig. S3). We next examined how long the backpacks remained on the cell surface: Flow cytometry suggested minimal loss within 6 h (Fig. S4), and confocal imaging showed stable surface attachment for at least 6 h, whereas obvious internalization was observed by 8 h (Fig. 2g). DMSNs were stably anchored on the ADSC surface via bioorthogonal click chemistry for at least 6 h, covering the critical early phase for oxidative stress protection and paracrine signaling. Subsequently, gradual internalization of DMSNs enables intracellular release of IGF-1, which can further activate survival and proliferative pathways such as PI3K/AKT and reinforce paracrine activity. This dual mechanism of surface-bound and internalized delivery ensures sustained therapeutic signaling, contributing to the enhanced regenerative performance of ADSC-IGF1@DMSN. To verify that backpack conjugation did not adversely affect the viability or function of ADSC, we evaluated cell viability across a range of grafting densities and no significant reduction in viability was observed (Figs. S5 and S6). Intracellular reactive oxygen species (ROS) levels were comparable before and after backpack conjugation, suggesting that the modification does not elicit oxidative stress in ADSCs (Fig. S7). In addition, we assessed the tri-lineage differentiation potential of ADSCs and confirmed that backpack decoration did not impair their differentiation capacity, indicating preserved stem cell functionality (Fig. S8). Collectively, these findings demonstrate that the IGF1@DMSN-ADSCs retain fundamental stem cell properties while acquiring a modular and stable nanoscale interface, thereby establishing a robust platform for subsequent functional enhancement and regenerative evaluation.

### Engineered Stem Cell Backpacks Reinforce ADSC Resilience and Regenerative Responsiveness under Oxidative Stress

**Fig. 3:**
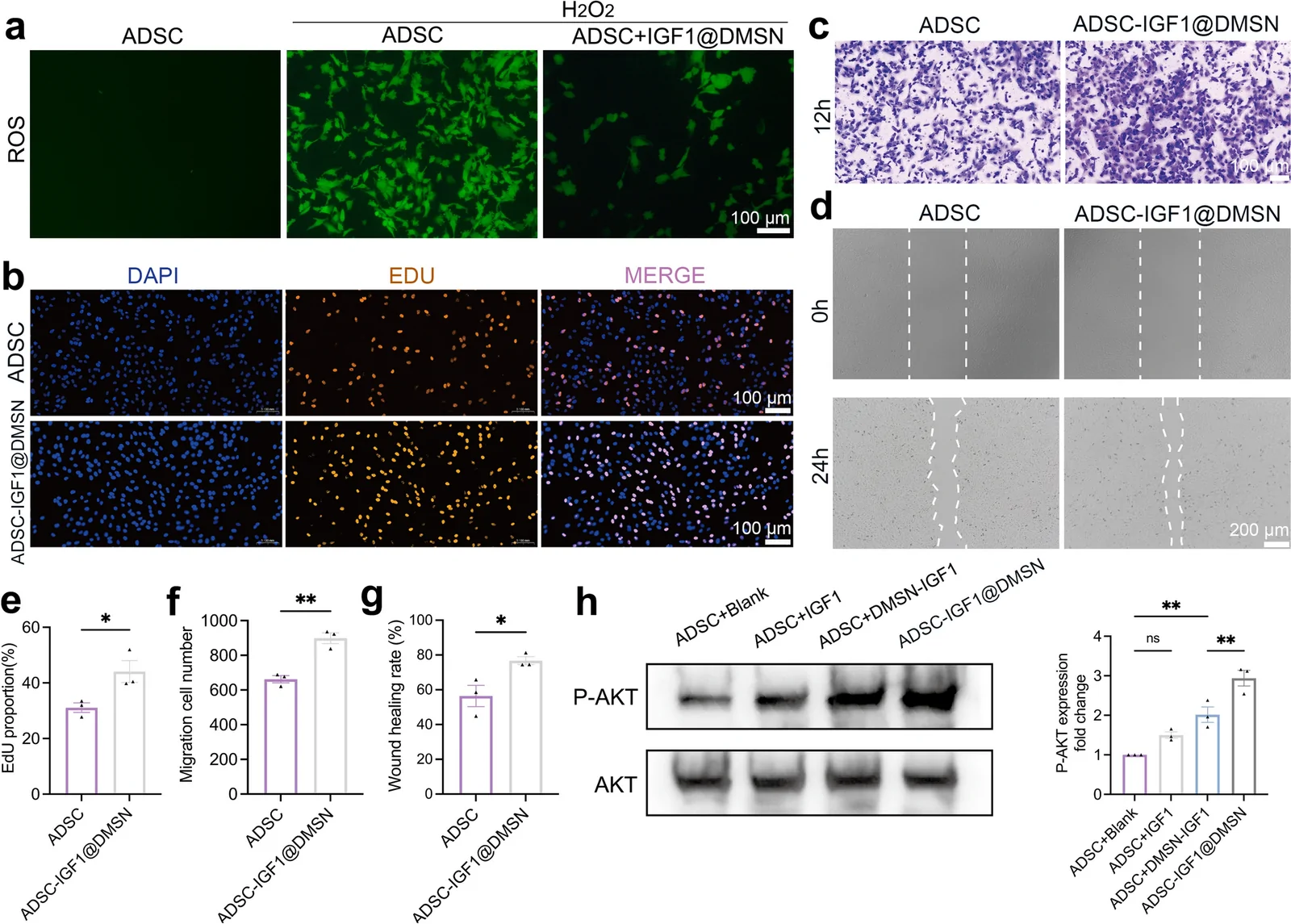
Engineered stem cell backpacks reinforce ADSCs microenvironmental resilience and regenerative behaviors. **a** Representative image of ADSC-IGF1@DMSN and ADSCs eliminating ROS under H₂O₂ stimulation. Scale bar, 100 μm. **b** Representative EdU staining images of ADSCs. Scale bar, 100 μm. **c** Representative images of migrated ADSCs in the two groups. Scale bar, 100 µm. **d** Representative image of scratch assay after 24 h among two different groups. Dotted lines indicate the interfaces. Scale bar, 200 µm. **e** Comparison of the relative number of proliferating ADSCs by EdU. **f** Comparison of the relative number of migrating cells by crystal violet staining. **g** Comparative analysis of wound healing rates. **h** Protein levels of Akt pathway-related markers in ADSCs from the two groups, and the comparison of Akt signaling pathway gene expression levels between two different groups. Student’s t-test or One-way analysis of variance (ANOVA) was performed for the comparison between groups. *n* = 3 (**e, f, g, h**)

After confirming the structural stability and biocompatibility of the backpack platform, we next investigated whether this interface engineering strategy could functionally reinforce ADSCs against hostile microenvironmental stress. Since oxidative stress is the primary factor limiting MSCs survival and their paracrine efficacy after transplantation, we first evaluated the ability of ADSC-IGF1@DMSN to reduce ROS levels under conditions of oxidative damage induced by hydrogen peroxide. Initial analysis of ADSCs by flow cytometry demonstrated positivity for stem cell markers such as CD44, CD73, and CD105 (Fig. S9). To elucidate the protective and enhancing effects of engineered modification on ADSCs proliferation and migration, we first established a H_2_O_2_ induced oxidative stress model to assess the ROS scavenging capacity of ADSC-IGF1@DMSN. ADSC-IGF1@DMSN effectively removed excess ROS (Figs. 3a and S10), indicating that the engineered nano-backpack endows ADSCs with enhanced microenvironmental resilience. In addition, we examined whether this cytoprotective effect was accompanied by improved regenerative cell performance. Compared with unmodified ADSCs, ADSC-IGF1@DMSN exhibited significantly enhanced proliferation (*P* < *0.05*; Fig. 3b, e). In parallel, transwell and scratch-wound assays consistently showed that engineered ADSCs possessed superior migratory activity relative to the ADSCs group (*P* < *0.05*; Fig. 3c, d, f, g). Consistently, the intracellular AKT signaling pathway was activated in ADSC-IGF1@DMSN as shown by western blot analysis (*P* < *0.05*; Fig. 3h), a pathway implicated in regulating cell survival, proliferation, and tissue repair. The results demonstrated that the backpack strategy does not merely preserve cell viability under oxidative stress, but actively strengthens core reparative behaviors of therapeutic cells. Altogether, these results support the central design principle of the platform, showing that surface-anchored nano-backpack protects ADSCs from hostile microenvironmental stress while enhancing their regenerative capacity. The dual functional reinforcement provides a mechanistic basis for the enhanced in vivo therapeutic efficacy observed in subsequent corneal repair studies.

### Engineered Stem Cell Backpacks Enhance Corneal Epithelial Resilience and Reparative Responses In Vitro

**Fig. 4:**
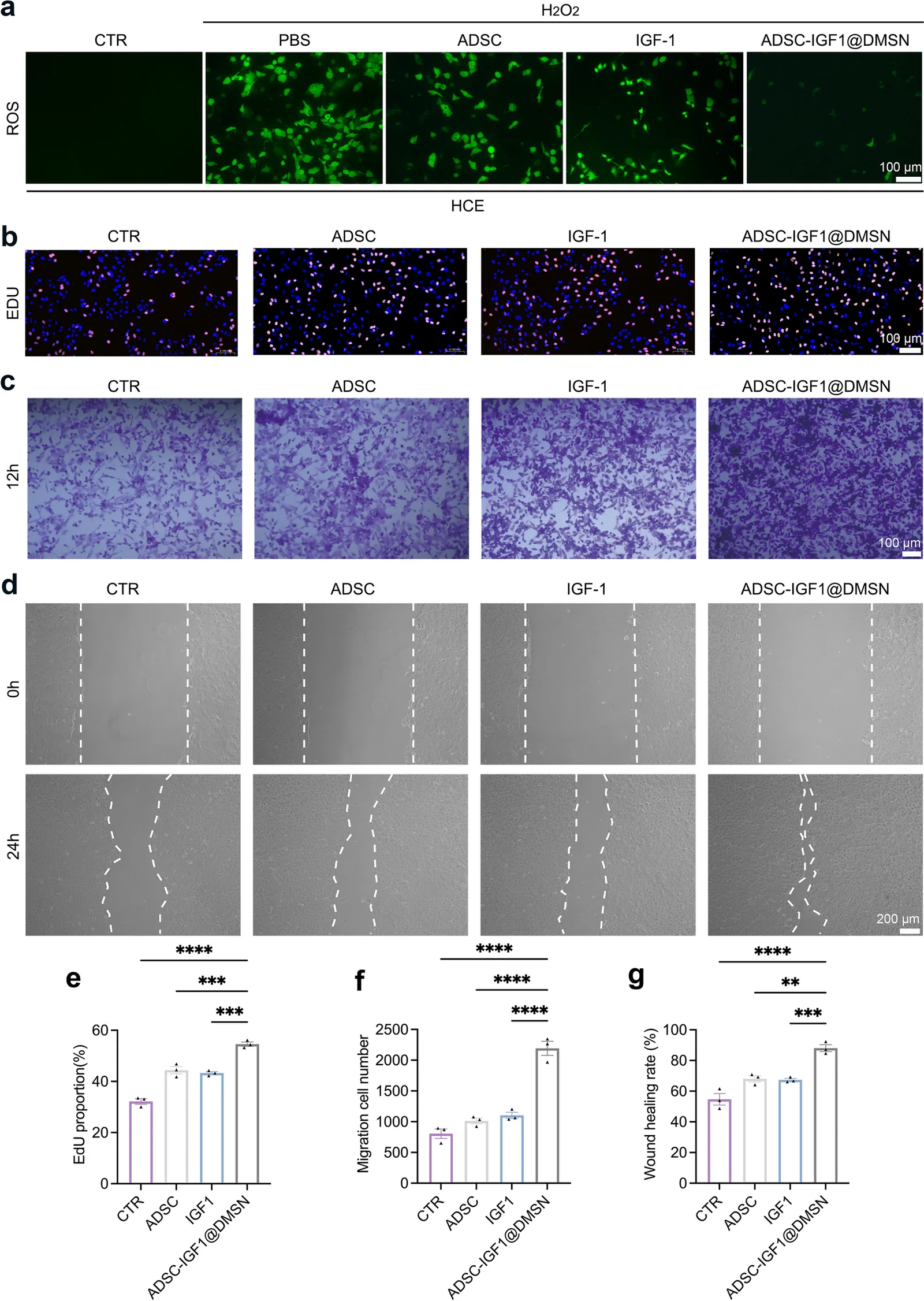
Engineered ADSC-IGF1@DMSN enhances corneal epithelial resilience and reparative responses in vitro. **a** Representative image of HCEs eliminating ROS under H₂O₂ stimulation across different treatment groups. Scale bar, 100 μm. **b** Representative EdU staining images of HCEs after 24 h co-culture. Scale bar, 100 μm. **c** Images of migrating HCEs obtained from Transwell assays depicting HCEs motility after 24 h of co-culture with ADSCs from the four groups. Scale bar, 100 µm. **d** Representative images of scratch assay after 24 h for four groups. Dotted lines indicate the interfaces. Scale bar, 200 µm. **e** Comparison of the relative numbers of proliferating HCEs by EdU. **f** Quantification of migrating cells based on crystal violet staining. **g** Comparative analysis of wound healing rates. One-way ANOVA was performed for comparison among the groups. *n* = 3 (**e, f, g**)

To confirm whether the stem cell backpack platform can transfer its protective and regenerative effects to injured epithelial target cells, we next examined the response of human corneal epithelial cells (HCEs) under conditions of oxidative stress and co-culture. Since excessive production of ROS is a primary cause of corneal epithelial damage, we first investigated the ROS scavenging capacity of ADSC-IGF1@DMSN in vitro using a cell model for oxidative stress induced by H_2_O_2_. Using DCFH-DA, we confirmed that ADSC-IGF1@DMSN effectively removed excess ROS within the HCEs (Figs. 4a and S11). ADSCs also showed some antioxidant activity, but its effect was less than that of ADSC-IGF1@DMSN, suggesting the engineered platform not only protects the carrier cells themselves, but also improves the local epithelial microenvironment through enhanced ROS buffering. We then assessed whether this improved microenvironmental conditioning could affect functional epithelial repair. We analyzed changes in the proliferation rate and migratory ability of HCEs after co-culture using a series of experiments. In particular, co-culture with ADSCs promoted the proliferation of HCEs, with HCEs in the ADSC-IGF1@DMSN co-culture group exhibiting the highest proliferation rate of all groups (*P* < *0.05*; Fig. 4b, e). Subsequently, the migratory ability of HCEs was examined under different co-culture conditions using Transwell and scratch assays. The results showed that ADSCs alone, IGF-1, and ADSC-IGF1@DMSN promoted the migration of HCEs, with the ADSC-IGF1@DMSN co-culture group showing the most significant improvement in migration (*P* < *0.05*; Fig. 4c, d, f, g). Since corneal epithelial healing following CCI depends both on the maintenance of epithelial viability and on active corneal re-epithelialization, these findings suggest that the backpack platform enhances the paracrine activity of ADSCs, thereby enhancing the reparative responsiveness of the corneal epithelium in vitro. To directly assess the paracrine effect of ADSC-IGF1@DMSN, conditioned media (CM) from each group were applied to HCEs. Scratch assays demonstrated that media from ADSC-IGF1@DMSN significantly accelerated wound closure compared with controls, and ELISA quantification revealed elevated HGF levels, confirming enhanced paracrine signaling (Fig. S12). Taken together, the in vitro findings in HCEs suggest that the bio-hybrid stem cell backpacks improve the damaged epithelial microenvironment by reducing oxidative stress and promoting epithelial proliferation and migration. This dual action bridges a critical mechanistic link between the engineered stem cell platform and its subsequent in vivo regenerative efficacy.

### Backpack Platform Drives Structural and Functional Recovery in a Rigorous CCI Proof-of-Concept Model

**Fig. 5:**
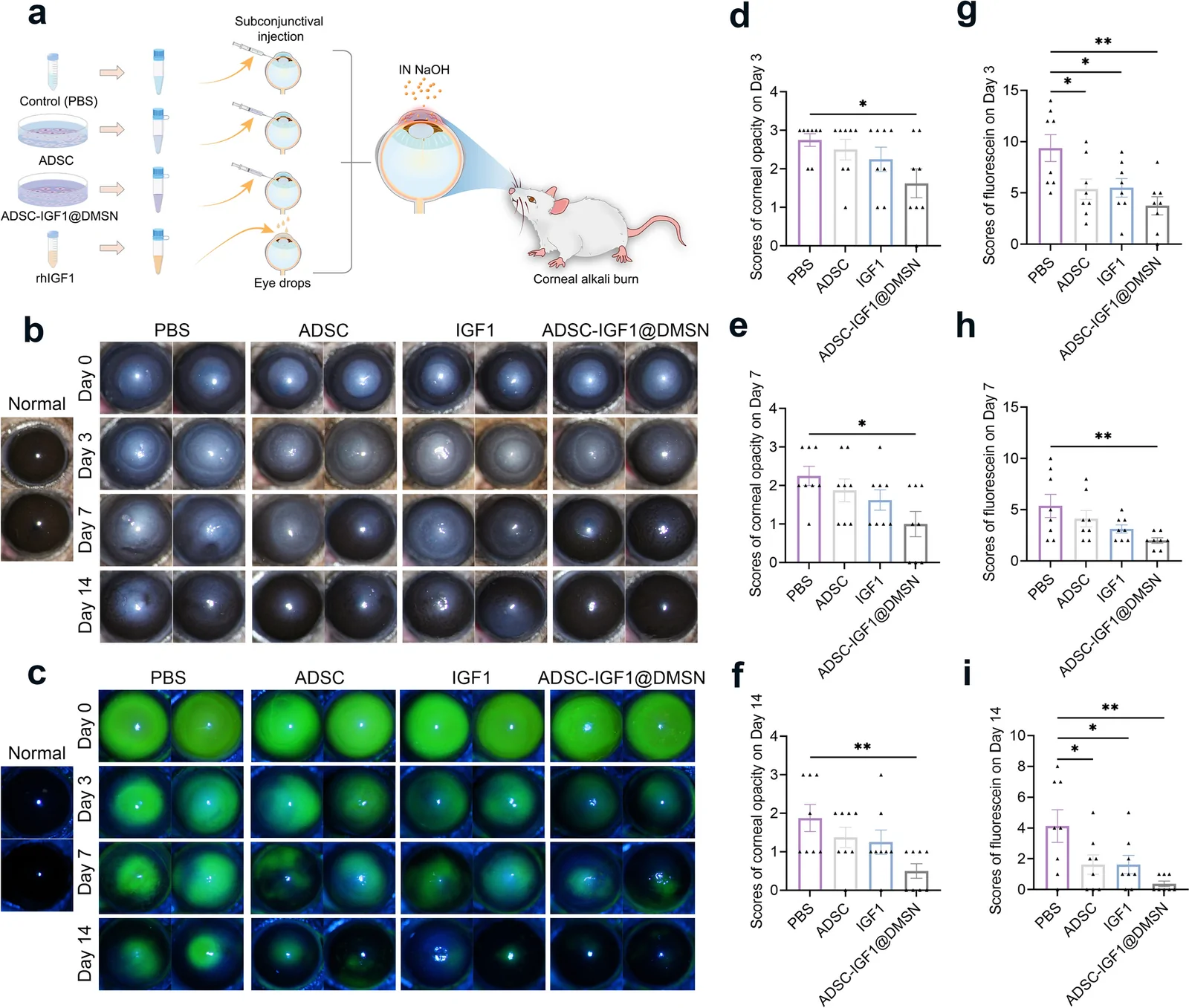
Stem cell backpack platform promotes structural and functional recovery in a corneal chemical injury proof-of-concept model. **a** Schematic illustration showing the establishment of the corneal chemical injury model and corresponding treatment strategies, including subconjunctival injection of PBS, ADSCs, or ADSC-IGF1@DMSN, whereas an additional group received topical IGF-1 eyedrops. **b** Representative images in the bright field at 0, 3, 7, and 14 days after chemical injury showing the dynamic changes in corneal opacity and transparency during corneal healing in normal and injured eyes. **c** Representative images obtained via fluorescein staining demonstrate the epithelial defect area and the progression of re-epithelialization across treatment groups at 0, 3, 7, and 14-day post-chemical injury. **d**–**f** Quantifications of corneal transparency scores at different time points. **g-i** Quantifications of fluorescein staining-based epithelial defect scores across different groups at 0, 3, 7, and 14-day post-chemical injury. One-way ANOVA was performed for comparison among the groups. *n* = 8 (**d–i**)

Having established that ADSC-IGF1@DMSN reinforces stem cell fitness and epithelial support in vitro, we next assessed whether this platform could deliver superior therapeutic benefit in vivo using CCI as a rigorous proof-of-concept model. A mouse model for CCI was developed to evaluate the therapeutic efficacy of ADSC-IGF1@DMSN. In addition to the normal control group, the injured mice were divided randomly into four treatment groups and administered subconjunctival injection or multiple topical administrations of phosphate-buffered saline (PBS), IGF-1, ADSC, or ADSC-IGF1@DMSN (Fig. 5a). Previous studies have shown that chemical injuries of the cornea compromise tear film stability and often lead to persistent dry eye, corneal erosion, and even corneal perforations in severe cases [20]. Therefore, we evaluated tear secretion using the phenol red test 14 days after the injury. The results showed that the ADSC-IGF1@DMSN group had a significantly higher tear volume than all other groups (Fig. S13). These results suggest that IGF-1, ADSC, and ADSC-IGF1@DMSN each restore tear film stability, with ADSC-IGF1@DMSN showing the strongest therapeutic effect.

To monitor corneal healing, corneal transparency was assessed using a slit-lamp microscope at 0, 3, 7, and 14 days after treatment (Fig. 5b). No significant differences were observed among groups of corneal transparency scores or fluorescein staining-based epithelial defect scores at Day 0 (Fig. S14). Quantitative analysis showed that the cornea treated with ADSC-IGF1@DMSN exhibited the highest degree of transparency and the greatest recovery (*P* < *0.05*; Fig. 5d–f). Specifically, compared with the group treated with ADSC alone, the ADSC-IGF1@DMSN group reduced corneal opacity from 1.375 to 0.5, with a 63.6% higher rate of improvement in corneal transparency than the ADSC group. Fluorescein sodium staining was used to evaluate epithelial defects (Fig. 5c), and the area of the epithelial defect and National Eye Institute (NEI) scores were analyzed on days 3, 7, and 14 *(P* < *0.05*; Fig. 5g–i*)*. Compared to the PBS group, all three treatments contributed to significantly accelerated epithelial regeneration. Interestingly, the cornea of the ADSC-IGF1@DMSN group achieved almost complete epithelial closure, with corneal morphology very similar to that of uninjured tissue. Compared with the ADSC treatment group, the engineered ADSC treatment group reduced the corneal defect score from 1.625 to 0.375, which is equivalent to a 76.9% improvement in corneal repair efficacy. Histological analysis confirmed these findings. Following chemical injury, partial detachment of corneal epithelial cells, mild edema of the connective tissue, and significant collagen disruption were observed (Fig. S15). Collectively, these data demonstrate that the bio-hybrid stem cell backpack platform substantially outperforms conventional factor delivery or stem cell monotherapy in this rigorous injury model, supporting the concept that active microenvironmental conditioning can translate into superior structural and functional recovery in vivo. A 14-day observation period was sufficient to assess the acute-to-subacute regenerative outcomes in our study, including epithelial closure, transparency restoration, barrier reconstruction, nerve regeneration, and suppression of inflammation and fibrosis. However, longer follow-up, such as 28 days, will be necessary in future studies to evaluate long-term tissue stability and biosafety.

### Backpack Platform Orchestrates Epithelial Barrier Reconstruction and Limbal Stem Cell Reactivation

**Fig. 6:**
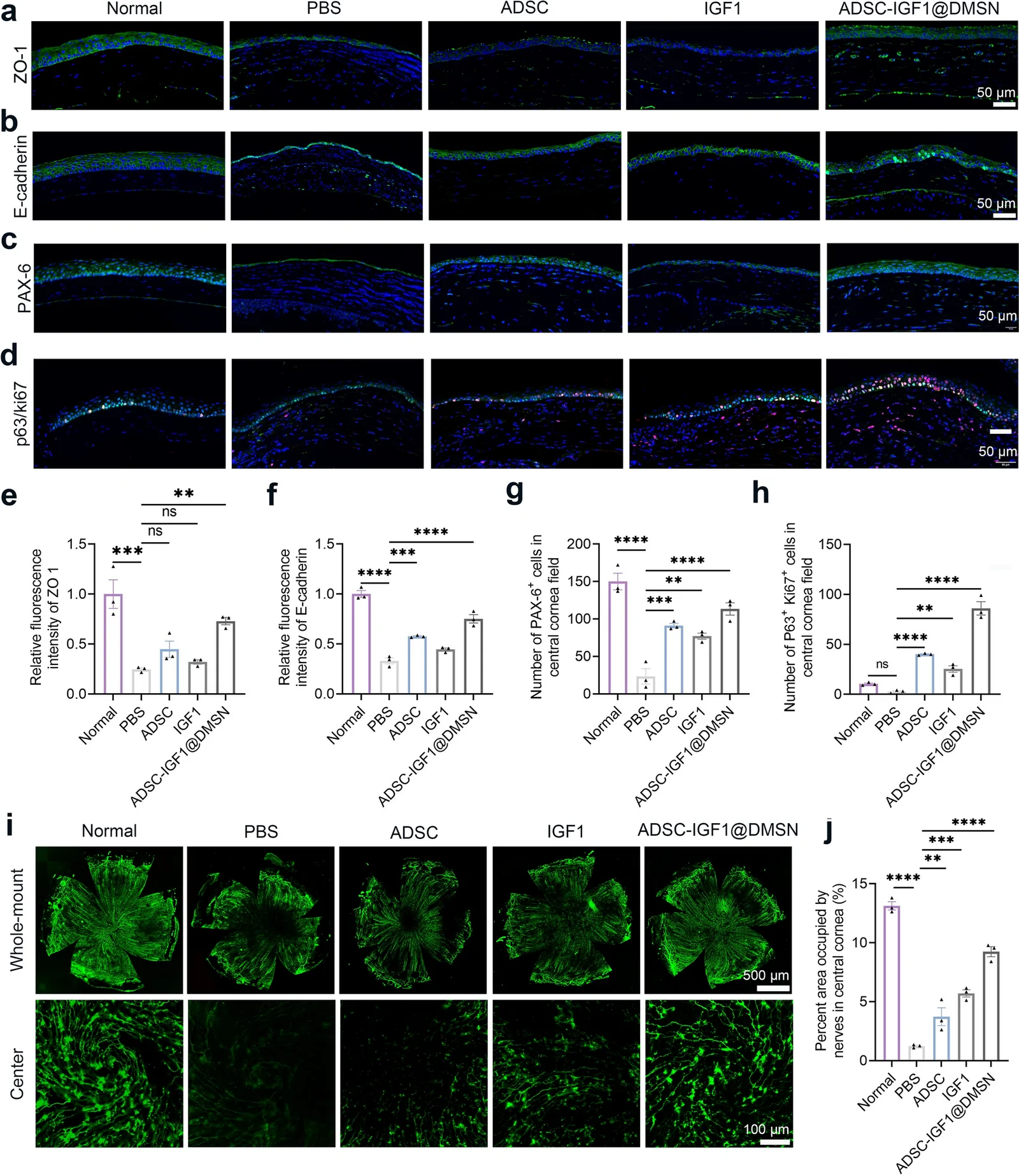
Backpack platform orchestrates epithelial barrier reconstruction, limbal stem cell activation, and corneal nerve regeneration after chemical injury. (**a**–**d**) Representative immunofluorescence images of corneal sections from the indicated groups stained for ZO-1 (a: tight-junction reassembly and barrier integrity), E-cadherin (b: epithelial cell–cell adhesion), PAX6 (c: corneal epithelial proliferation and differentiation), and p63/Ki67 (d: the activity and proliferative response of LSCs). Scale bar, 50 μm. **e** Relative fluorescence intensity of ZO-1 expression from each group was compared.** f** Relative fluorescence intensity of E-cadherin expression from each group was compared**. g** Quantification of PAX6^+^ cells per field in corneas of each group.** h** Quantification of p63^+^ Ki67^+^ per field in corneas of each group.** i** Representative images of whole cornea of tubulin b-III staining and the respective images of central corneal nerve fibers within each group are shown. Scale bar, 500 μm and 100 μm. **j** Quantification of central corneal nerve coverage, representing the percentage areas covered by nerve fibers, with comparisons across groups. One-way ANOVA was performed for comparison among the groups. *n* = 3 (**e**–**h**, **j**)

To clarify how ADSC-IGF1@DMSN restores corneal surface integrity, we next examined epithelial barrier markers and regenerative epithelial programs in injured corneas. Immunohistochemical staining was performed using the ZO-1 marker for the tight junctions of the cornea [36]. The cornea after treatment with ADSC-IGF1@DMSN showed a distinct and continuous ZO-1 staining pattern, similar to that observed in a normal cornea, while the other groups showed a weaker and discontinuous staining pattern. These results suggest that treatment with ADSC-IGF1@DMSN helps maintain corneal epithelial barrier integrity (*P* < *0.05*; Fig. 6a, e). In addition, we also examined the E-cadherin expression, another important marker of the epithelial barrier [37]. The results revealed that treatment with ADSC-IGF1@DMSN markedly promoted the recovery of corneal epithelial barrier function (*P* < *0.05*; Fig. 6b, f). These findings demonstrate that the engineered platform not only accelerates wound closure macroscopically, but also promotes functional barrier reconstruction on the tissue level.

We then investigated whether this structural repair was accompanied by activation of corneal epithelial regenerative programs. The expression and distribution of PAX6, an important transcription factor for maintaining the balance and properties of the corneal epithelium, were analyzed [38]. Staining of the entire cornea showed a significant increase in PAX6-positive cells in the ADSC-IGF1@DMSN-treated group (*P* < *0.05*; Fig. 6c, g). Most epithelial cells showed strong PAX6 expression in the cell nucleus. This result suggests that the engineered system specifically activates epithelial cells to promote corneal regeneration. In comparison, corneas from the PBS group exhibited significantly reduced PAX6 fluorescence intensity, indicating impaired epithelial integrity.

With the rapid advancement of stem cell biology, it is widely recognized that LSCs are critical for corneal epithelial repair and regeneration [39, 40]. LSCs mainly reside within the basal epithelial layer of the limbal region, where the cornea and sclera meet. Under normal physiological conditions, these stem cells remain largely inactive to maintain epithelial homeostasis. However, when exposed to environmental influences or injuries to the cornea, LSCs are activated and undergo symmetric or asymmetric cell division to initiate regeneration of the epithelium [41–43]. Through coordinated migration to the center of the cornea and stepwise differentiation, LSCs continuously regenerate corneal epithelial cells, ensuring dynamic epithelial renewal, tissue homeostasis, corneal transparency, and the maintenance of long-term visual function. P63 acts as a marker of LSCs in basal layers, while Ki67 indicates proliferating cells. Based on this, we examined the cell proliferation activity in basal layers using immunofluorescence double staining for P63 and Ki67. Compared to the other three injured groups, the group treated with ADSC-IGF1@DMSN exhibits a considerably higher number of P63⁺/Ki67⁺ cells in limbal basal layers **(P** < *0.05*; Fig. 6d, h**).** Together, these results indicate that the backpack platform promotes corneal repair at multiple levels, including barrier reassembly, epithelial lineage maintenance, and endogenous stem cell reactivation. Such coordinated regulation is consistent with the concept that ADSC-IGF1@DMSN orchestrates multidimensional regeneration rather than delivering a single function wound healing effect.

### Engineered Platform Supports Corneal Nerve Reinnervation as Part of Multidimensional Regeneration

True functional recovery after CCI requires restoration not only of the epithelial surface but also of corneal innervation, which is essential for trophic support, epithelial homeostasis, and long-term ocular surface stability. To clarify the morphological distribution of corneal nerve fibers after injury and different treatments, fluorescent immunostaining of the entire cornea for tubulin β-III and whole cornea photography were performed. Representative images of corneal nerve fibers at the center of the chemical lesion showed that ADSC-IGF1@DMSN treatment plays a crucial role in corneal nerve regeneration and recovery after chemical injury (Fig. 6i). Quantitative analysis of nerve fibers in middle cornea demonstrated that the nerve fiber density in the ADSC-IGF1@DMSN-treated group was significantly greater than all other treatment groups (*P* < *0.05*; Fig. 6j). Corneal nerves secrete neurotransmitters, which are critical factors for maintenance of epithelial homeostasis [44]. The damage to corneal nerves causes a decrease in trophic support and disrupts the balance of the epithelium. In addition, neurotransmitters and proinflammatory cytokines induce apoptosis in the epithelium and weaken the cornea’s support for nerve regeneration [45]. Ultimately, the cascade leads to degeneration and dysfunction of the corneal nerves, thereby exacerbating ocular surface disorders. The robust reinnervation observed after ADSC-IGF1@DMSN treatment therefore suggests that the platform interrupts this degenerative feedback loop and reestablishes a regenerative microenvironment favorable for neuroepithelial recovery. In the context of the overall study, nerve regeneration further underscores that the therapeutic benefit of this system extends beyond surface closure to coordinated restoration of multiple functional corneal compartments.

### ADSC-IGF1@DMSN Suppresses Pathological Angiogenic and Lymphangiogenic Remodeling

**Fig. 7:**
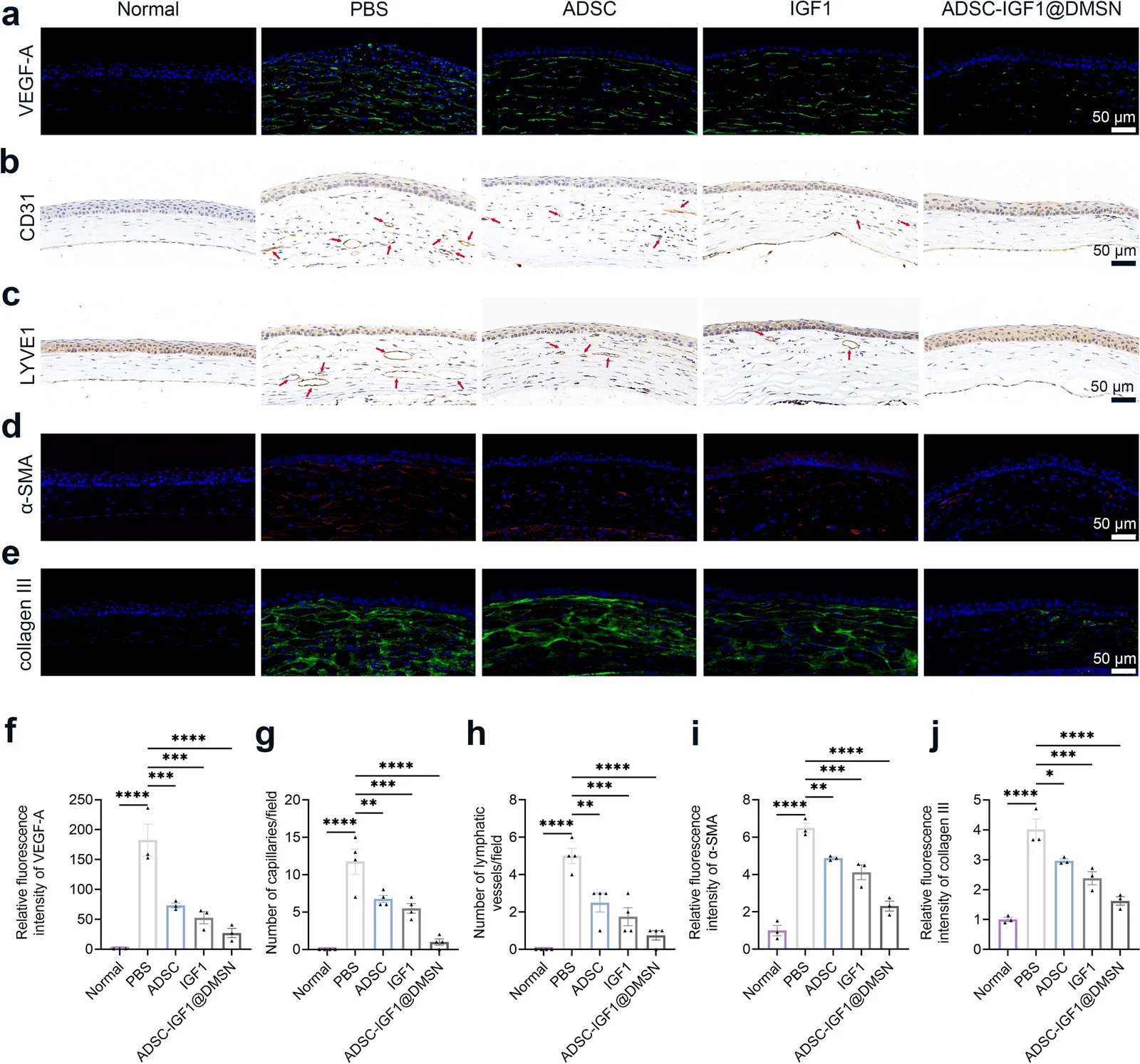
Backpack platform suppresses pathological angiogenic, lymphangiogenic, and fibrotic remodeling in injured corneas. **a** Representative immunofluorescence image of VEGF-A in cornea from the designated group, reflecting the pro-angiogenic signaling after injury and treatments. Scale bar, 50 μm. **b, c** Representative immunohistochemical staining images for CD31(b; blood vessel endothelium) and LYVE1 (c; lymphatic endothelium). The red arrow denotes representative micro-vessels and lymphatic structures. Scale bar, 50 μm. **d** A representative immunofluorescence image of α-SMA to assess myofibroblast activation in corneal tissues from the indicated groups. Scale bar, 50 μm.** e** Representative immunofluorescence staining images of Collagen Ⅲ to assess fibrotic responses in corneas from each group. Scale bar, 50 μm.** f** Quantification of VEGF-A fluorescence intensity from each group. **g, h** Quantitative of CD31^+^ capillary density and LYVE1.^+^ lymphatic vessel density per field among groups.** i** Relative fluorescence intensity of α-SMA expression from each group was compared.** j** Relative fluorescence intensity of Collagen Ⅲ expression from each group was compared. One-way ANOVA was performed for comparison among the groups. *n* = 3 (**f**, **i**, **j**), *n* = 4 (**g**, **h**)

Since persistent inflammation after CCI commonly drives pathological neovascularization and lymphangiogenesis, we next examined whether the backpack platform could restrain this maladaptive remodeling. Immunohistochemical staining for VEGF-A showed strong staining in the cornea of the PBS group, while it was significantly attenuated after treatment with ADSC-IGF1@DMSN (*P* < *0.05*; Fig. 7a, f). Immunohistochemical analysis of the CD31 and LYVE1 revealed that treatment with ADSC-IGF1@DMSN significantly suppressed the formation of new capillaries and lymphatic vessels in comparison with the PBS group (Fig. 7b, c). The mean number of new capillaries in the PBS group was 11.75 ± 3.40/visual field, while it was only 1.00 ± 0.82/visual field in the ADSC-IGF1@DMSN group (*P* < *0.05*; Fig. 7g). Similarly, the density of lymphatic vessels decreased from 5.00 ± 0.82/visual field in the PBS group to 0.75 ± 0.50/visual field in the ADSC-IGF1@DMSN-treated cornea (*P* < *0.05*; Fig. 7h). Treatment with ADSC alone or with IGF-1 eyedrops also resulted in a partial reduction in capillary and lymphatic vessel formation, but the effect was less pronounced. These data demonstrate that ADSC-IGF1@DMSN more effectively restrains inflammatory vascular remodeling, which is critical for preserving corneal avascularity and optical clarity after injury.

### ADSC-IGF1@DMSN Attenuates Fibrotic Remodeling and Preserves Corneal Transparency

In addition to abnormal vessel growth, excessive inflammatory responses not only induce new blood vessel formation in the cornea, but also promote corneal fibrosis. The result is a rapid deterioration of vision, which in severe cases can lead to blindness [46]. PBS-treated corneas showed strong expression of α-SMA (a marker of myofibroblasts) and type III collagen (a marker of excessive extracellular matrix deposition) (Fig. 7d, e). In contrast, α-SMA and collagen III expression was significantly reduced in all treated groups, with the greatest suppression of fibrosis observed in the ADSC-IGF1@DMSN group (*P* < *0.05*; Fig. 7i, j). These antifibrotic effects are mechanistically and functionally meaningful. By suppressing stromal scarring, the engineered platform helps preserve the structural basis of transparency, which is consistent with the superior slit-lamp recovery and lower opacity scores observed in the same group (Fig. 5b). Thus, beyond improving epithelial healing, ADSC-IGF1@DMSN also suppresses long-term maladaptive remodeling, further supporting its role as an active niche-modulating therapeutic platform rather than a passive cell delivery vehicle.

### ADSC-IGF1@DMSN Remodels the Inflammatory Niche through Suppression of NF-κB/MAPK Signaling

**Fig. 8:**
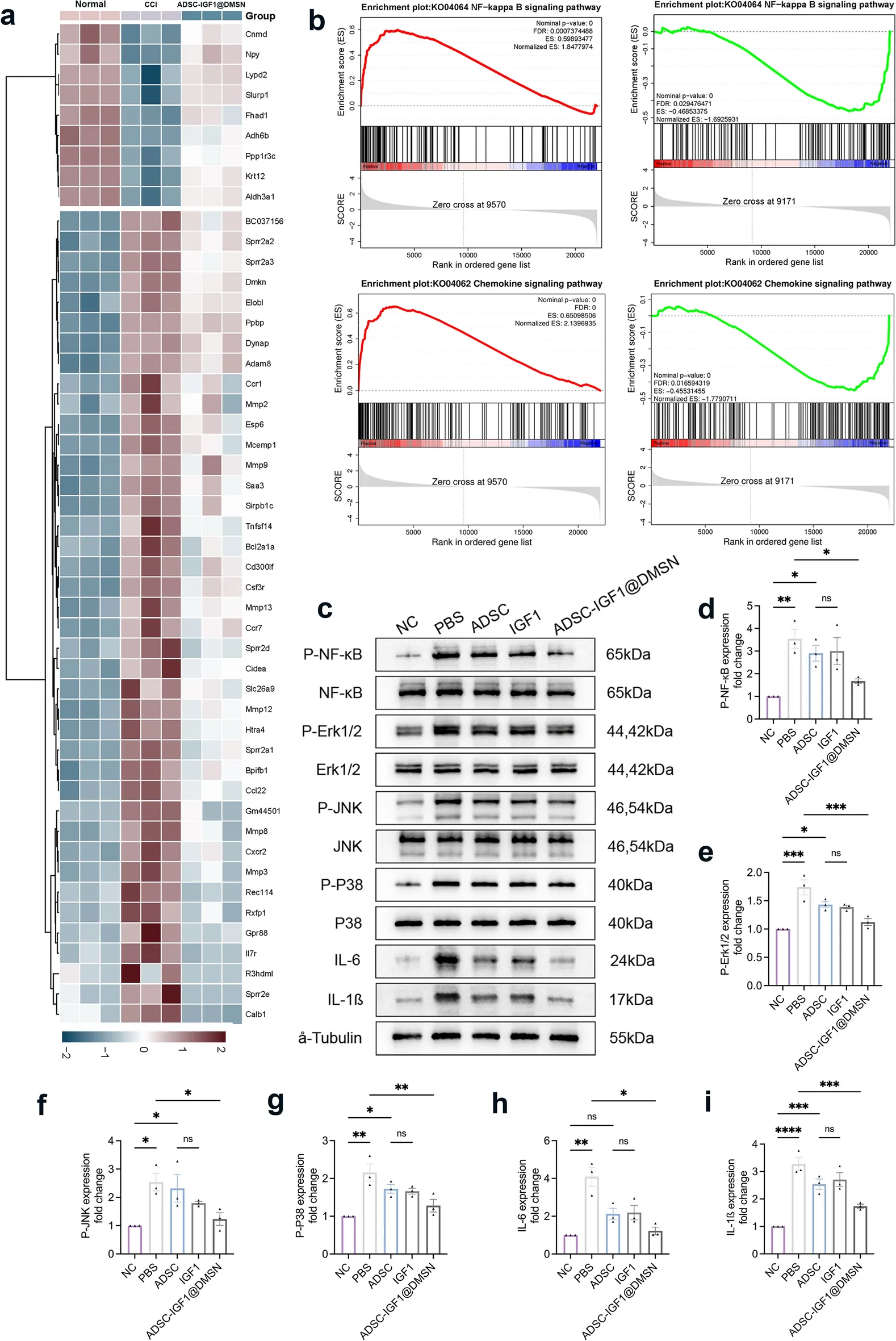
Backpack platform remodels the inflammatory niche and promotes regeneration through suppression of NF-κB/MAPK signaling. **a** Heatmap of transcriptomic sequencing results from normal mice, PBS-injected CCI mice, and ADSC-IGF1@DMSN-injected CCI mice.** b** Gene set enrichment analysis (GSEA) for gene sets altered between the ADSC-IGF1@DMSN group and PBS group.** c** Protein levels of NF-κB/MAPK signaling pathway and inflammatory markers in corneas among all treatment groups. **d-i** Quantitative analysis of NF-κB/MAPK signaling pathway and inflammatory gene expression levels among all treatment groups. One-way ANOVA was performed for comparison among the groups. *n* = 3 (**d**–**i**)

To gain mechanistic insight into how the engineered platform coordinates these broad regenerative outcomes, we performed transcriptomic analysis of corneas from normal mice, PBS-treated CCI mice, and ADSC-IGF1@DMSN-treated CCI mice. Hierarchical cluster analysis identified genes related to inflammation, fibrosis, and neovascularization (Ccr1, Ccr7, Cxcr2, Ccl22, Mmp9, Mmp13, Saa3, and Tnfsf14) after CCI, but all of these genes were significantly reduced after treatment with ADSC-IGF1@DMSN (Fig. 8a). The suppression of chemokine receptors (Ccr1, Ccr7, and Cxcr2) suggests reduced leukocyte mobilization and inflammation [47], while reduced metalloproteinase levels (Mmp2, Mmp3, Mmp9, and Mmp13) suggest extracellular matrix (ECM) remodeling and suppression of excessive fibrosis [48–50]. In addition, genes associated with acute inflammation and programmed cell death regulation (Saa3, Tnfsf14) were also downregulated [51–53], confirming that ADSC-IGF1@DMSN reduces inflammation in the cornea and promotes an optimal balance for healing. Meanwhile, the high expression of Calb1 (calbindin 1), the calcium-binding protein involved in the protection and regeneration of nerve cells [54, 55], suggests the activation of neuroprotective signaling pathways that could promote corneal nerve recovery. Gene set enrichment analysis revealed that the NF-κB and chemokine signaling pathways are highly activated in the CCI cornea but are significantly inhibited after treatment with ADSC-IGF1@DMSN (Fig. 8b). GO analysis revealed enrichment of biological processes related to neurogenesis, chemotaxis, angiogenesis, and stem cell differentiation, indicating that ADSC-IGF1@DMSN simultaneously modulates inflammation and activates endogenous regenerative programs (Fig. S16). The findings indicate that ADSC-IGF1@DMSN does not act through a single downstream target, but instead broadly reprograms the post-injury niche toward a regenerative state.

We then validated this inflammatory remodeling at the protein level. CCI caused strong phosphorylation of NF-κB p65, and MAPK signaling, which was significantly reduced after treatment, especially in the ADSC-IGF1@DMSN group (Fig. 8c). Consistent with this, IL-1β and IL-6 levels, which had previously increased significantly after injury, also decreased (*P* < *0.05*; Fig. 8d-i). These results indicate that ADSC-IGF1@DMSN treatment is associated with downregulation of NF-κB/MAPK inflammatory signaling and improved corneal repair, concomitant with attenuated pathological vascular and fibrotic remodeling, as well as enhanced epithelial regeneration, stem cell activation, and nerve reinnervation. In this sense, ADSC-IGF1@DMSN functions as an active microenvironmental modulator that converts an inflammatory, degeneration-prone niche into a pro-regenerative therapeutic landscape. It should be noted, however, that in the absence of specific NF-κB/MAPK modulation experiments, the observed changes in these signaling pathways can only be interpreted as correlative rather than causative. Future studies employing pathway-specific inhibition or activation will be necessary to directly elucidate the role of NF-κB/MAPK signaling in ADSC-IGF1@DMSN-mediated corneal repair.

#### In Vivo Safety Evaluation

After subconjunctival injection of ADSCs and engineered ADSCs for 14 days, we collected major organs from the mice for toxicity assessment. H&E staining revealed no histological abnormalities in the eyeball, heart, liver, spleen, lung, or kidney tissues of the injection group compared to the control group. CD45 immunohistochemical staining further showed that none of the treatment groups exhibited increased immune cell infiltration in eyeballs compared with the control group (Fig. S17). In addition, the biochemical indicators RBC, WBC, HGB, PLT, ALT, AST, BUN, and CREA were all within normal ranges (Fig. S18). While our study assessed short-term safety in major organs and corneal tissues, MSNs are known to gradually degrade into soluble silicic acid in vivo and are primarily excreted renally [56]. Their systemic accumulation is minimal at doses comparable to those used here, with negligible long-term toxicity reported. These properties support the favorable biosafety of the ADSC-IGF1@DMSN platform and its potential applicability in other regenerative therapies. In summary, the engineered ADSCs exhibited excellent biocompatibility in vivo and did not induce any detectable toxicity.

## Conclusion

In summary, we report a universal bio-hybrid stem cell backpack platform that enables precise cell surface nanoengineering to convert mesenchymal stem cells into active therapeutics for microenvironmental modulation. By mildly anchoring IGF-1-loaded dendritic mesoporous silica nanoparticles onto ADSCs through bioorthogonal chemistry, this strategy endows the engineered cells with two synergistic properties, namely enhanced microenvironmental resilience and sustained paracrine signaling, thereby overcoming key barriers that constrain stem cell therapy in regenerative medicine. In a rigorous corneal chemical injury model, ADSC-IGF1@DMSN suppresses ROS accumulation and NF-κB/MAPK-associated inflammatory signaling, reshapes a pro-regenerative niche, and promotes multidimensional tissue repair, including epithelial regeneration, restoration of transparency, corneal nerve reinnervation, limbal stem cell activation, and inhibition of fibrosis, neovascularization, and lymphangiogenesis. Combined with its favorable in vivo biocompatibility and modular, cargo-interchangeable design, this platform defines a versatile and scalable therapeutic framework for diverse tissue injuries that face similar microenvironmental challenges. More broadly, this study highlights a conceptual shift from passive stem cell delivery to active programming of the injury microenvironment.

## Supplementary Information

Below is the link to the electronic supplementary material.

Supplementary file1 (DOCX 4185 kb)
