# Supplementary material for: A Universal Bio-Hybrid Nanoparticle Backpack Platform Endows Stem Cells with Microenvironmental Resilience and Sustained Paracrine Signaling
Source: Nanomicro Lett. 2026 Jul 20;18:441. doi: 10.1007/s40820-026-02308-3 (PMC13385731; doi:10.1007/s40820-026-02308-3)
Supplement: Supplementary file 1 — Supplementary file1 (DOCX 4185 kb) [file 40820_2026_2308_MOESM1_ESM.docx]

Supporting Information for

**A Universal Bio-Hybrid Nanoparticle Backpack Platform Endows Stem Cells with Microenvironmental Resilience and Sustained Paracrine Signaling**

Yuqing Chen^1,†^, Ying Yang^2,†^, Shuo Yang^1,†^, Xingyi Shu^1^, Zhiyong Liu^1^, Jian Song^1^, Ya-Xuan Zhu^3,*^, Han Lin^3,4,*^, Ruili Wei^1,*^, Jianlin Shi^3,4^

^1^ Department of Ophthalmology, Shanghai Changzheng Hospital, Second Affiliated Hospital of Naval Medical University, Shanghai 200003, P. R. China

^2^ Department of Ophthalmology, Wuxi No. 2 People's Hospital, Wuxi 214000, P. R. China

^3^ Shanghai Tenth People's Hospital, Shanghai Frontiers Science Center of Nanocatalytic Medicine, School of Medicine, Tongji University, Shanghai 200072, P. R. China

^4^ State Key Laboratory of High Performance Ceramics, Shanghai Institute of Ceramics Chinese Academy of Sciences, Shanghai 200050, P. R. China.

^†^Yuqing Chen, Ying Yang, and Shuo Yang contributed equally to this work.

* Corresponding authors. E-mail: [ruiliwei@smmu.edu.cn](mailto:ruiliwei@smmu.edu.cn) (Ruili Wei); [linhan@mail.sic.ac.cn](mailto:linhan@mail.sic.ac.cn) (Han Lin); [yxzhu1994@tongji.edu.cn](mailto:yxzhu1994@tongji.edu.cn) (Ya-Xuan Zhu)

**S1 Experimental Section**

**S1.1 Materials**

TEOS, CTAB, NaSal, APTES, and Chol-PEG-FITC were purchased from Aladdin Chemistry Co., Ltd. (Shanghai, China). HCl, methanol, and ethanol were obtained from Sinopharm Chemical Reagent Co., Ltd. (Shanghai, China). NHS-PEG-DBCO, NHS-PEG-N_3_ and IGF-1 were obtained from MCE, NHS-Cy5 was purchased from Shanghai Yuanye Bio-Technology Co., Ltd. Hoechst 33342 was obtained from (Beyotime Biotechnology (Shanghai, China).

**S1.2 Synthesis and characterization of DMSN**

DMSNs were synthesized according to a previously reported method with minor modifications. To be specific, 68 mg of TEA were added to 25 mL deionized water and stirred gently at 80℃ for 0.5 h. Subsequently, 380mg CTAB and 168mg NaSal, serving as structural directing agent and silica source, respectively, were added to the above solution and kept stirring for another 1 h. Then, 4 mL TEOS was added with gentle stirring (~ 300 rpm) for 12 h. The products were collected centrifugation (20000 rpm, 10 min) and washed several times with ethanol to remove the residual reactants. To remove the surfactant template, the precipitate was extracted in an HCl/methanol solution at 6℃ for 6 h, repeated three times. The obtained DMSNs were finally dried under vacuum at room temperature overnight. To introduce amine groups onto the DMSN surface, DMSNs (50 mg) and APTES (50 µL) were dispersed in ethanol (100 mL) and reacted under reflux at 78℃ for 12 h.

The DMSN-DBCO was synthesized via the reaction between DMSN-NH2 and NHS-PEG-N_3_ via amide coupling reaction. To be specific, 5 mg DMSN-NH_2_ and 0.25 mg NHS-DBCO were reacted in phosphate buffer (PB, pH 7.4) under room temperature for 4 h, and then washed with deionized water for 3 times.

To load IGF-1 into the DMSN-DBCO, DMSN-DBCO and IGF-1 were mixed in deionized water at a weight ratio of 1000:1, and kept stirring at 4℃ overnight. We washed the product with PBS for 3 times to remove unloaded IGF-1.

The hydrodynamic sizes and zeta potentials of DMSN, DMSN-NH_2_, and DMSN-DBCO were measured via a zetasizer (Nano AS90, Malvern Instrument). The morphology and elemental distribution were characterized via transmission electron microscopy (TEM, JEM-2100F, JEOL). The encapsulation efficiency of IGF-1 was measured by ELISA.

**S1.3 Preparation and characterization of ADSC-IGF1@DMSN**

ADSCs were first harvested by trypsinization and washed once with PBS. The cells were then incubated with NHS-PEG-N_3_ (10 µg/mL) at room temperature for 15 min. After centrifugation, the azide-functionalized ADSCs were subsequently incubated with IGF1@DMSN-DBCO at room temperature for 20 min, followed by centrifugation (1500 rpm, 5 min, 4 ℃) and washed by PBS twice to remove unbound backpacks.

To demonstrate the efficient surface conjugation of IGF1@DMSN, we labeled DMSN with NHS-Cy5 and coupled it onto the ADSCs following the method described above. For SEM imaging, the ADSCs-IGF@DMSN were fixed overnight and dehydrated before SEM imaging. Then the cells were stained with Hoechst 33342 (10 µg/mL, 10 min) and Chol-PEG-FITC (2 µg/mL, 5 min) before confocal imaging. To assess backpack retention and internalization, confocal images were acquired at indicated time points after conjugation. The flow cytometry was also used to quantify the fluorescence signals of ADSCs. The cell viabilities of ADSC-IGF1@DMSN with different DMSN concentration were evaluated via cell counting kit-8 (cck-8) assay. The ROS levels of ADSC conjugated with IGF1@DMSN were also measured.

**S1.4 Isolation of Primary ADSCs**

This research was approved by Changzheng Hospital of Naval Medical University. Animal experiments and protocols were approved by the Medical Ethics Committee of Shanghai Changzheng Hospital (License No.2025SLYS1). Eight weeks old male C57BL/6 mice were obtained from Shanghai Jihui Biotechnology. ADSCs were isolated according to previously reported protocols [S1]. After euthanasia, we incised the skin at the inguinal region to expose and isolate adipose tissue, which was transferred to a culture dish which contained prechilled phosphate-buffered saline (PBS). We digested collected tissue at 37°C with 0.1% collagenase I (Gibco, Carlsbad, CA) for 30 minutes. After centrifugation, we seeded the cells and cultured them at 37°C incubator with 5% carbon dioxide, with the medium being replaced every two days. ADSC cells were passaged when they reached a confluence rate of 80 to 90%. Cells that had been passaged three times were used for the following experiments.

**S1.5 Phenotypic Characterization of ADSCs**

To evaluate surface marker expression, 5 × 10⁴ ADSCs were incubated at room temperature for 30 minutes using fluorophore-conjugated antibodies against CD44, CD73, and CD105 (BD Biosciences, San Jose, California). The low cytometry was analyzed with FlowJo software (Tree Star, Ashland, Oregon). The experiments were all repeated three times.

**S1.6 Tri-Lineage Differentiation of ADSCs**

The ADSCs’ ability of adipogenic, osteogenic, and chondrogenic differentiation potential was evaluated using a standard stimulation medium (OriCell, Saijie Bio, China) according to manufacturer's protocol. ADSC-IGF1@DMSN were cultured in induction mediums for 21 days and fixed with 4% paraformaldehyde. The differentiation potential into adipocytes, osteoblasts, and chondrocytes was ascertained by Oil RedO, Alizarin RedS, and Alcian Blue staining, respectively. The stained cells were photographed using an optical microscope.

**S1.7 Reactive Oxygen Species (ROS)**

HCEs were cultured overnight in a 12-well plate (1×10⁴ cells). They were then treated for 4 hours with ADSC, IGF-1, or ADSC-IGF1@DMSN. After washing with PBS, they were treated with 400 μM H₂O₂ for 45 minutes to induce temporary excessive ROS in the cells. Following three washings with PBS, we incubated the cells in the dark for 30 minutes with the DCFH-DA probe (Beyotime Biotechnology, China). ROS levels in the cells were assessed by microscope.

**S1.8 EdU**

Cell proliferation was assessed using a 5-ethynyl-2'-deoxyuridine (EdU) incorporation assay. After 24 hours of co-culture of HCEs with ADSCs or ADSC-IGF1@DMSN, an EdU solution (1:1000 dilution) was incubated for 2 hours. Cells were then fixed for 15 minutes using 4% paraformaldehyde, washed for 5 minutes with glycine solution, and twice with 0.3% Tween X-100. They were incubated at room temperature in the dark with Apolloazol (one-click reaction solution) for 30 minutes, then three washes with 0.3% Triton X-100. The cell nuclei were stained with DAPI for 30 minutes and subsequently washed three times with PBS. The stained cells were photographed using fluorescence microscope. The cell numbers were measured using ImageJ software.

**S1.9 Cell Migration Assay**

Cell migration capacity was evaluated by 24-well polycarbonate transwell inserts (Thermo Fisher Scientific, USA). The HCEs (2 × 10³) was seeded in the upper chamber, while ADSC cells and ADSC-IGF1@DMSN cells were seeded in the lower chamber to demonstrate the effect on HCEs migration. After 24 hours of co-culture, we fixed the cell in 4% paraformaldehyde (PFA) for 15 minutes and stained with 0.1% crystal violet solution for 20 minutes. The migrated cells were observed on three randomly selected areas under an optical microscope and counted using ImageJ. To evaluate the direct impact of engineered modifications on ADSC migration, 2 × 10³ cells were seeded in the upper chambers, with the addition of 200 μL medium. Simultaneously, we added 600 μL medium into the lower chamber. And after incubation for 24 hours, the cells were fixed, stained, and measured as described above.

**S1.10 Scratch Wound Healing Assay**

We seeded HCEs (5 × 10⁵) in 6 well plates and cultured to confluence. Twenty-four hours of coculture with ADSC-IGF1@DMSN, a linear wound was generated with sterile 200 μL pipette tips. We rinsed with PBS for three times. To inhibit proliferation, we then add fresh medium to continue the cell culture. At specific time points, we photographed the wound closure under the microscope and analyzed the migration distance using ImageJ software.

**S1.11 Establishment of a Corneal Chemical Injury Model**

The procedure for establishing chemical corneal lesion models was based on previous reports. The specific procedure is as follows: After general and local anesthesia, Whatman III filter papers (Whatman, UK) saturated in 2 μL of 1N NaOH solutions were placed on central corneas for 20 seconds and then rinsed with PBS for 1 minute.

**S1.12 In vivo safety assessment**

An in vivo safety assessment was performed 14 days after injection of ADSC-IGF1@DMSN. First, a hematoxylin and eosin (H&E) staining and CD45 immunohistochemical staining were conducted. Tissue sections from eyeballs, hearts, livers, spleens, lungs, and kidneys were sectioned into 10 μm thick slices and underwent to H&E staining. Whole blood samples were collected and routine blood tests were performed.

**S1.13 Classification of animals and clinical evaluation**

Firstly, we randomly divided the mice into five groups, including normal control, PBS, ADSC, IGF-1 eye drop, and ADSC-IGF1@DMSN group. Each group consisted of 8 mice. The PBS group received a subconjunctival injection of 10 μL of PBS. The ADSC group received a subconjunctival injection of 10 μL cell suspension containing 1×10⁵ ADSC. The IGF-1 groups accept 1 mg/ml rhIGF-1 four times per day for 14 days. The ADSC-IGF1@DMSN group received a subconjunctival injection of 10 μL cell suspension containing 1×10⁵ ADSC-IGF1@DMSN. All injections were performed under anesthesia using a 10 μL syringe (Hamilton, USA) with a 33-gauge metal needle.

We performed the clinical examinations at predefined times, and the researchers involved in data evaluation and analysis were unaware of group assignments. We examined the corneas using the slit lamp microscope (Suzhou Liuliu Vision Technology Co., Ltd., China) to assess corneal opacity, epithelial healing, and edema. The assessment times included the period before treatment (day 0) and several times after treatment. Corneal opacity was assessed using a specific classification system. Level 0 corresponds to total transparency; level 1 corresponds to slight opacity with clear visibility of the iris and pupil; level 2 corresponds to moderate opacity with recognizable iris and pupil; level 3 corresponds to severe opacity with difficulty recognizing the iris and pupil; level 4 corresponds to total opacity where the iris and pupil are not visible. The corneal epithelial defect was examined using the cobalt blue light with 1% sodium fluorescein staining (Sigma-Aldrich). The images were analyzed and surface areas of epithelial defects were measured using the National Eye Institute (NEI) scoring system. This scoring system divides the cornea into five zones, each scored from 0 to 3 points, with a maximum of 15 points.

Tear secretion was evaluated by phenol thread test, in accordance with the procedure described above [S2]. Briefly, we placed a phenol red thread (Jing Ming, Tianjin, China) in the lateral conjunctiva fornix for 15 seconds, and the length of the wet thread (in mm) was measured. Six eyes were randomly selected from each group and subjected to the tear secretion test.

**S1.14** **Histology and Immunohistochemistry**

At day 14 post-injury, H&E and Masson’s trichrome staining were performed according to standard methods. As for immunohistochemistry, we blocked the sections using 5% donkey serums one hour, after overnight incubation at 4 °C using the primary antibody against CD31 or LYVE1 (Servicebio, China). After rinsing, secondary antibody (1:2000, Servicebio) was used for 1 hour at RT. After acquiring the images, we analyzed it by ImageJ.

**S1.15 Immunofluorescence Staining**

After 15 minutes of incubation with 0.3% Triton X-100, the membrane was blocked for one hour at RT using 5% donkey serum. The primary antibody against PAX6 (Servicebio), α-SMA (Servicebio), collagen III (Servicebio), p63 (Servicebio), Ki67 (Servicebio), ZO-1 (Servicebio), and E-cadherin (Servicebio) were then applied for overnight incubation at 4 °C. On the following day, we incubated it for one hour with fluor-conjugated secondary antibody, then counterstained with DAPI for 10 minutes. Fluorescence intensity was measured using ImageJ.

**S1.16 Corneal Nerve Staining**

The whole corneal sample was fixed for one hour in paraformaldehyde (PFA), then blocked for two hours. We incubated it overnight at 4°C using anti-β-III tubulin antibody (Abcam), followed by 1 hour incubation with the secondary antibody. We cut the cornea radially and photographed by the fluorescence microscope. Nerve fibers density was assessed using ImageJ software.

**S1.17 Western Blot**

The total proteins were isolated from cultured cells or corneal tissue utilizing a digestion buffer containing phosphatase and protease inhibitors. The protein concentrations of the cells or tissues were assessed by BCA kit. Proteins were separated by 10% SDS-PAGE gel electrophoresis and transferred into PVDF membranes. And after blocking membrane for one hour at RT by TBST containing 5% milk powders, we incubated it overnight at 4°C with α-TUBULIN, IL-1β, IL-6, ERK, JNK, P38, NF-κB p65, Akt (total protein and phosphorylated form, CST). And after washing, membrane was incubated by a secondary antibody (Sigma-Aldrich) at room temperature for 1 hour. We visualized protein bands via an enhanced chemiluminescence (ECL, Thermo Fisher Scientific) and quantified by ImageJ software.

**S1.18 Statistical Analysis**

All data were reported as mean ± standard deviation (SD). The statistical analysis was conducted by GraphPad Prism software. Depending on the experimental design, the comparisons were performed by the Student's t-test, and ANOVA. p-value < 0.05 was regarded ad statistically significant.

**S2 Supplementary Figures**


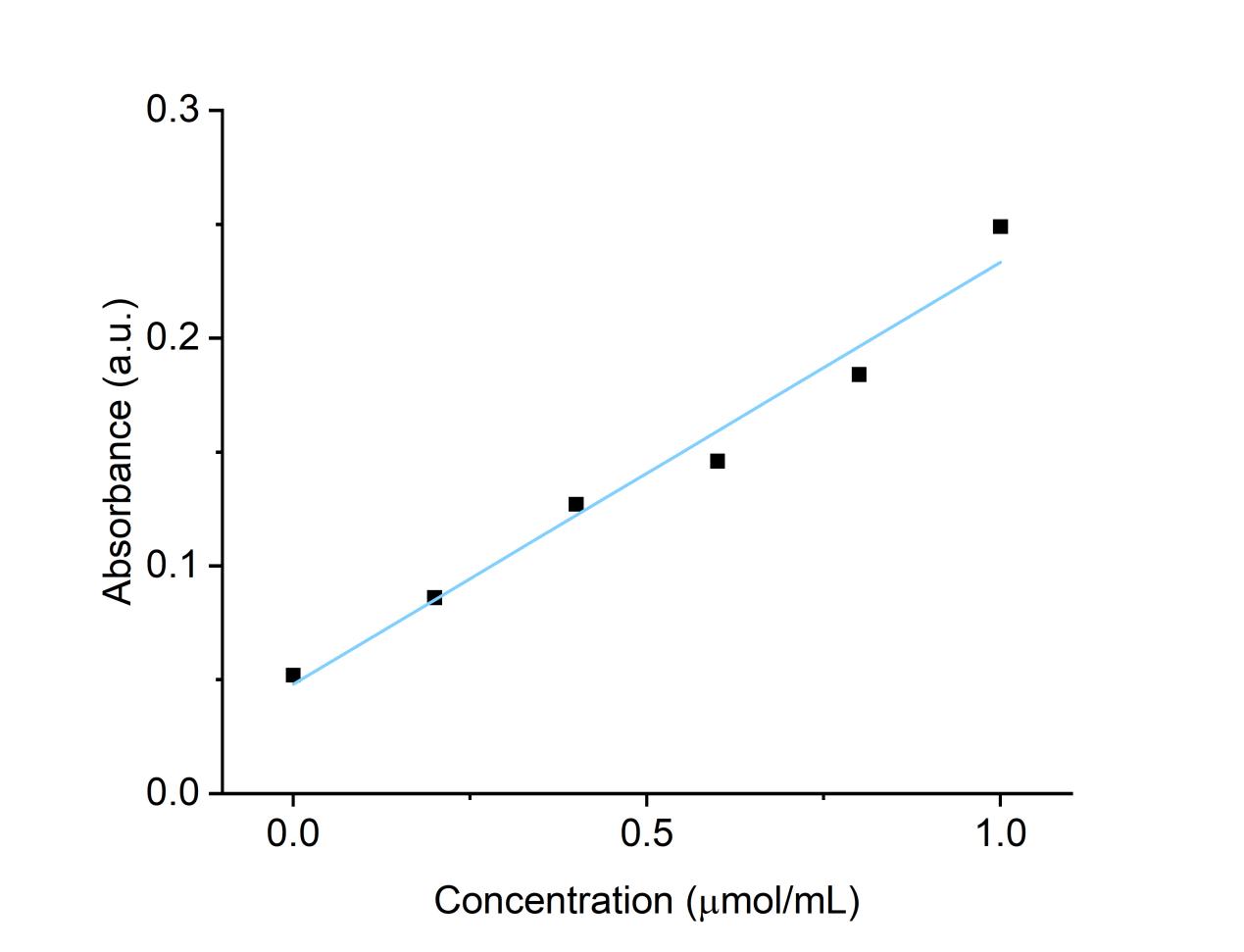


**Fig. S1** Calibration curve of the ninhydrin assay using a primary amine standard (570 nm), used to quantify surface -NH_2_ groups.


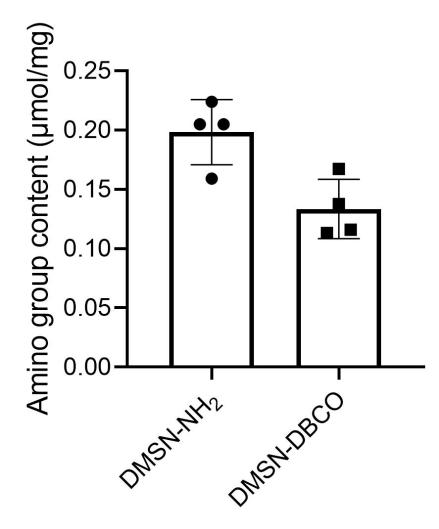


**Fig. S2** Surface -NH_2_ contents on DMSN-NH_2_ and DMSN-DBCO. n = 4.


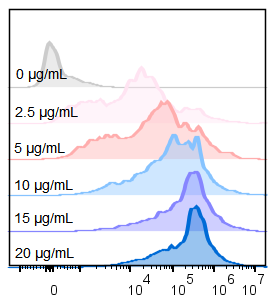


**Fig. S3** Flow cytometry results of ADSCs decorated with different concentrations of Cy3-labeled DMSN-DBCO.


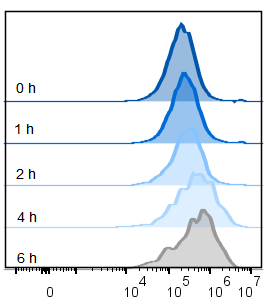


**Fig. S4** Time dependent fluorescence intensity changes of ADSC-DMSN measured by flow cytometry, DMSNs were labeled with Cy3.


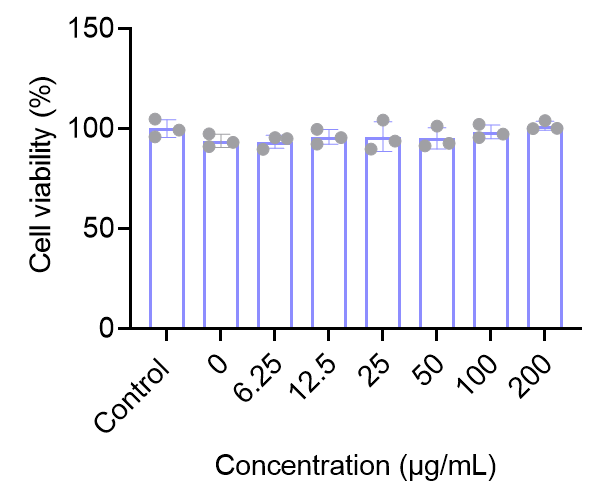


**Fig. S5** Cell viabilities of ADSCs first treated with NHS-PEG-N3 (10 µg/mL), and subsequently incubated with different concentrations of DMSN-DBCO. n = 3.


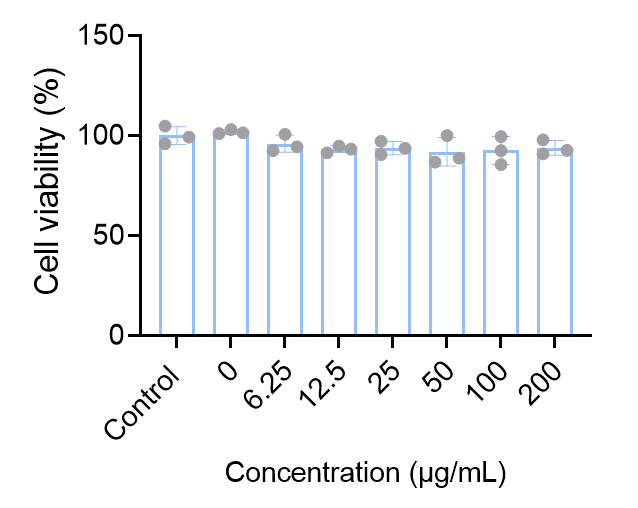


**Fig. S6** Cell viabilities of ADSCs first treated with NHS-PEG-N3 (25 µg/mL), and subsequently incubated with different concentrations of DMSN-DBCO. n = 3.


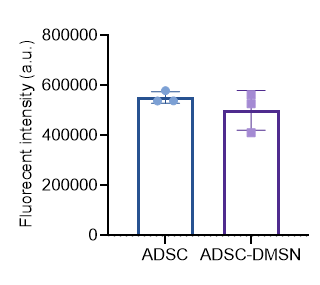


**Fig. S7** Fluorescence intensity of ADSC or ADSC-DMSN stained with DCFH-DA measured by flow cytometry. n=3.

**
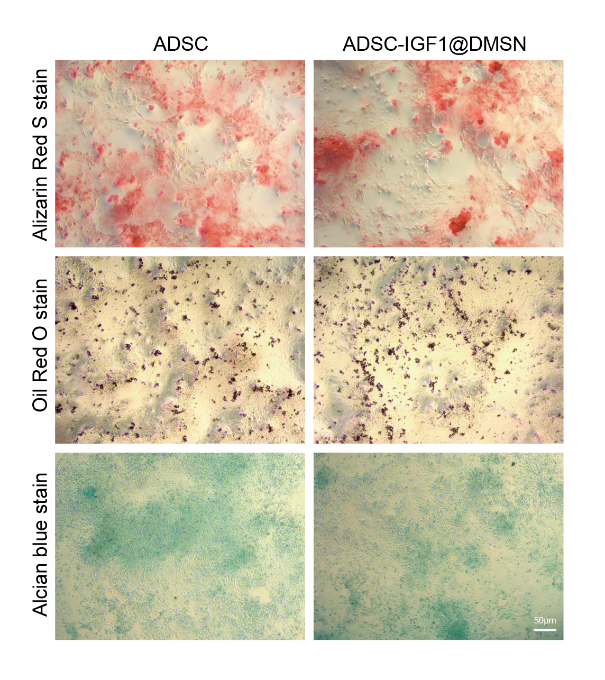
**

**Fig. S8** Triple lineage differentiation capability of engineered ADSC-IGF1@DMSN.


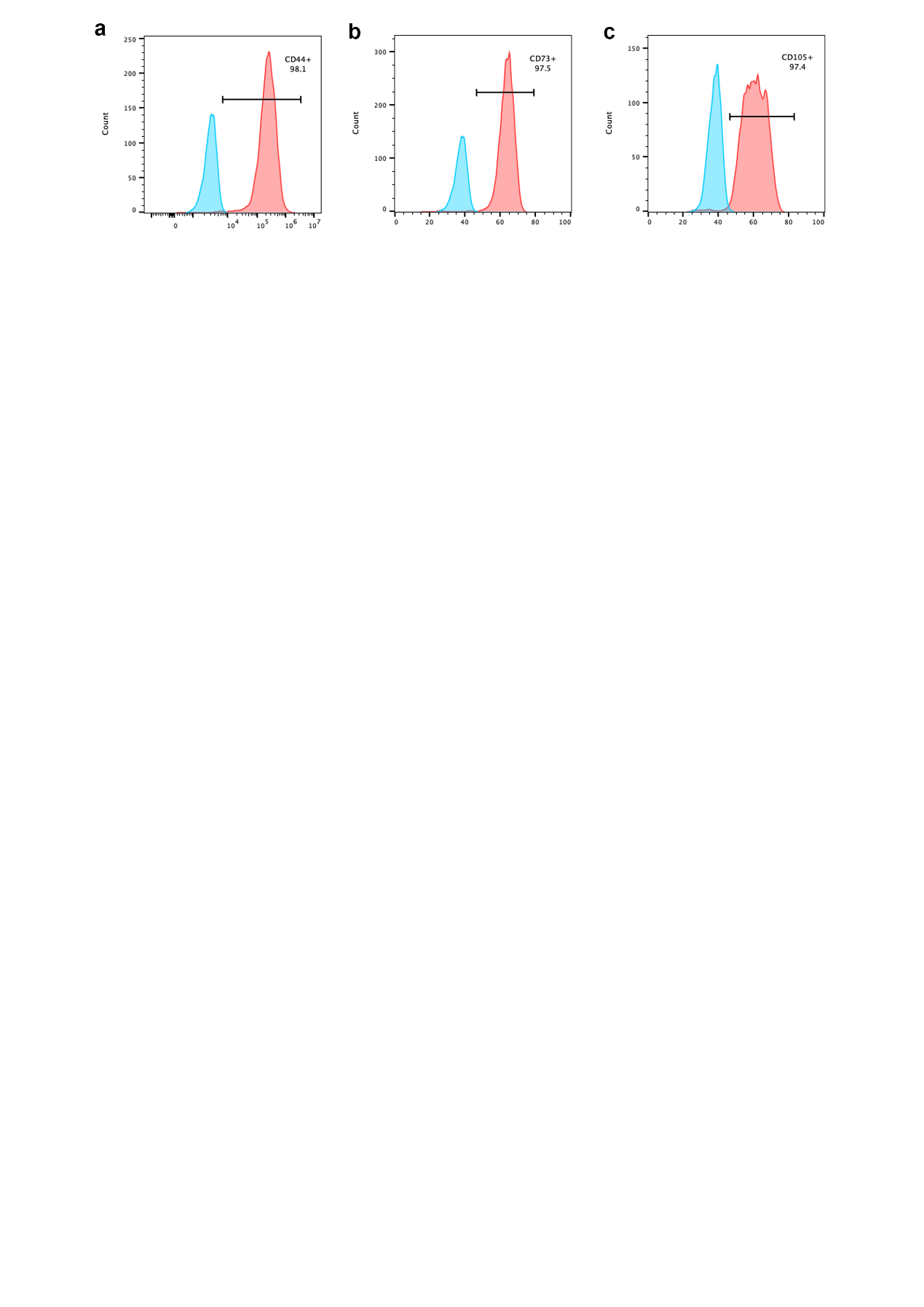


**Fig. S9** Flow Cytometry Identification of Primary ADSCs.

**
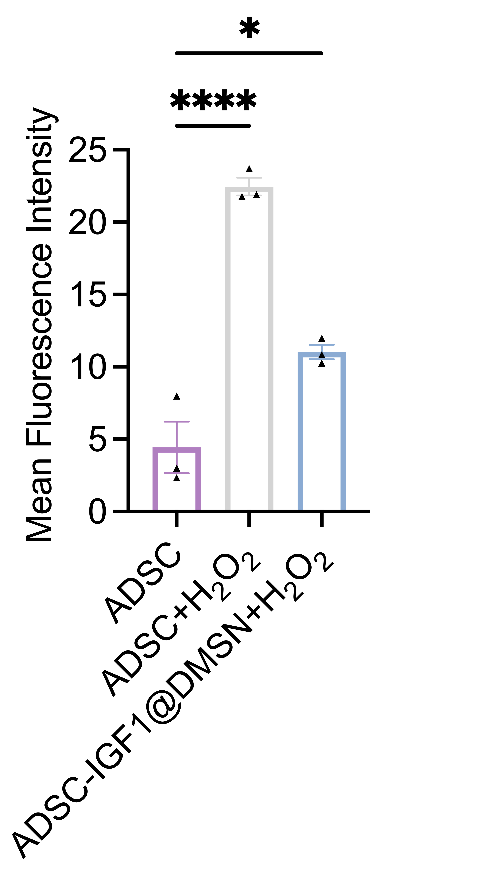
**

**Fig. S10** Quantitative analysis of mean ROS fluorescence intensity in each group. One-way ANOVA was performed for comparison among the groups, n = 3.

**
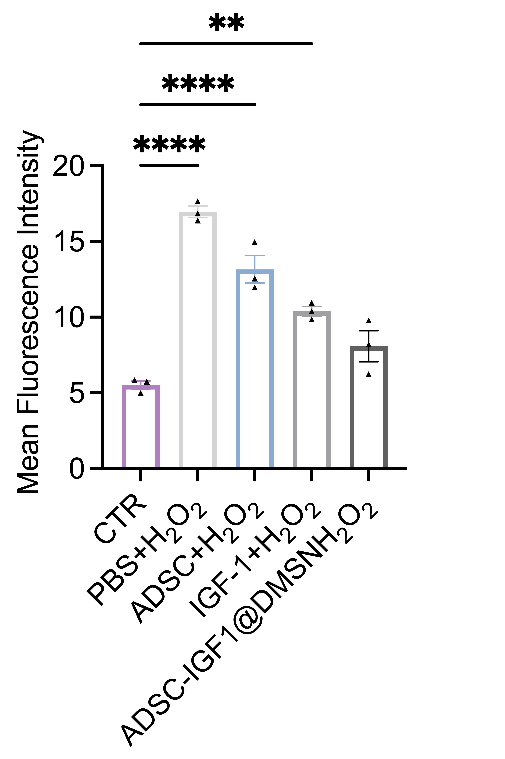
**

**Fig. S11** Quantitative comparison of mean ROS fluorescence intensity in HCEs under H₂O₂ stimulation with different treatments. One-way ANOVA was performed for comparison among the groups, n = 3.


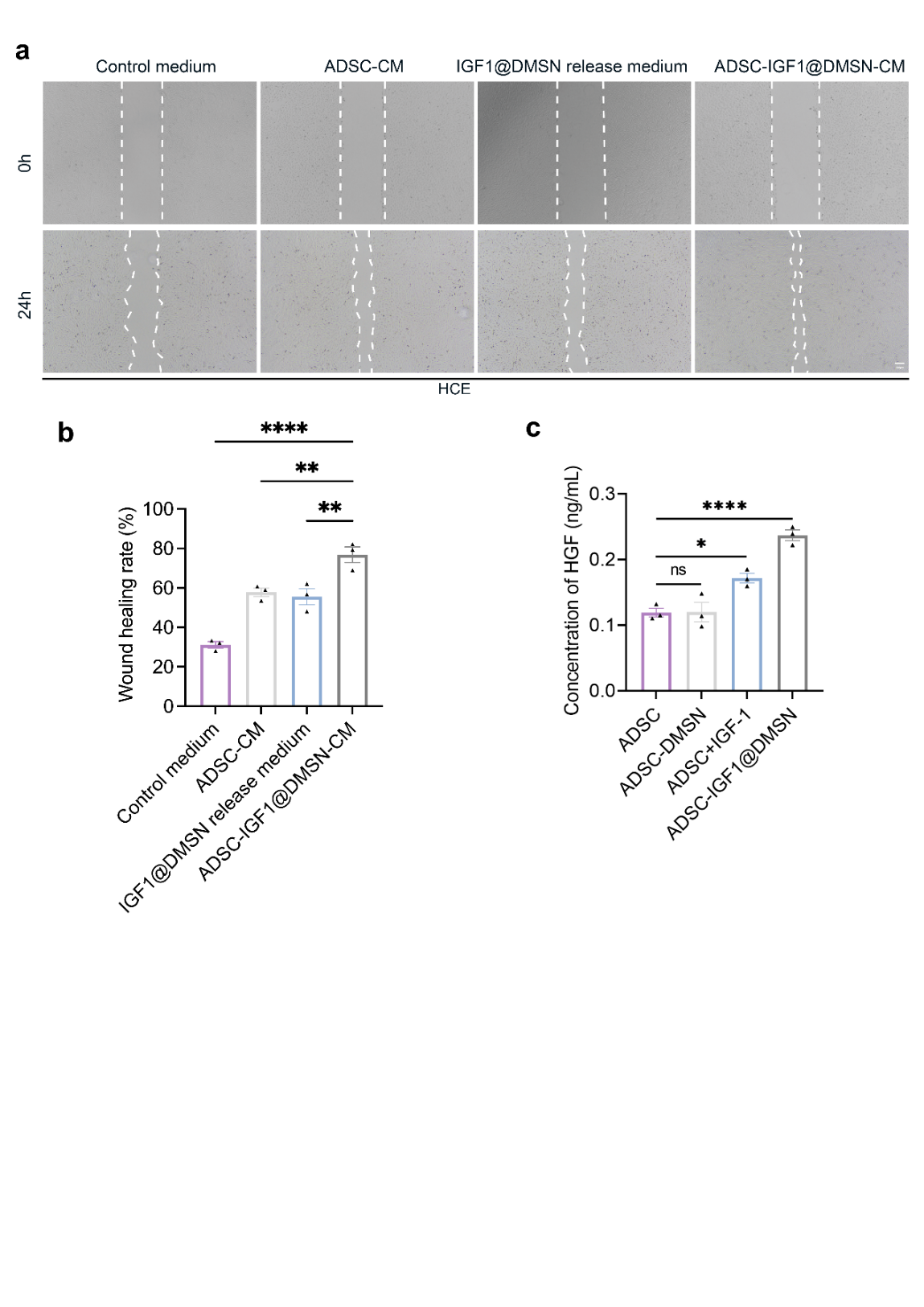


**Fig. S12 a** Representative images of scratch assay after 24 hours for four groups. Dotted lines indicate the interfaces. Scale bar, 100 µm. **b** Comparative analysis of wound healing rates. **c** ELISA measurement of HGF concentrations in the conditioned media (CM) from each group of cells. One-way ANOVA was performed for comparison among the groups. n = 3 (b, c).


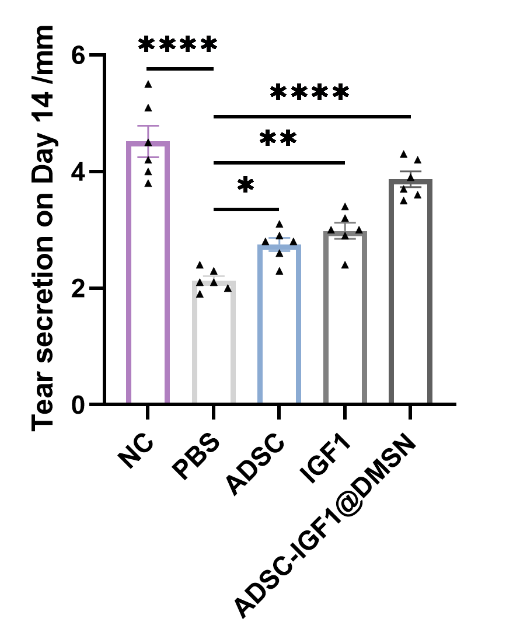


**Fig. S13** Tear secretion volume in mice after treatment in each group. One-way ANOVA was performed for comparison among the groups. n=6.


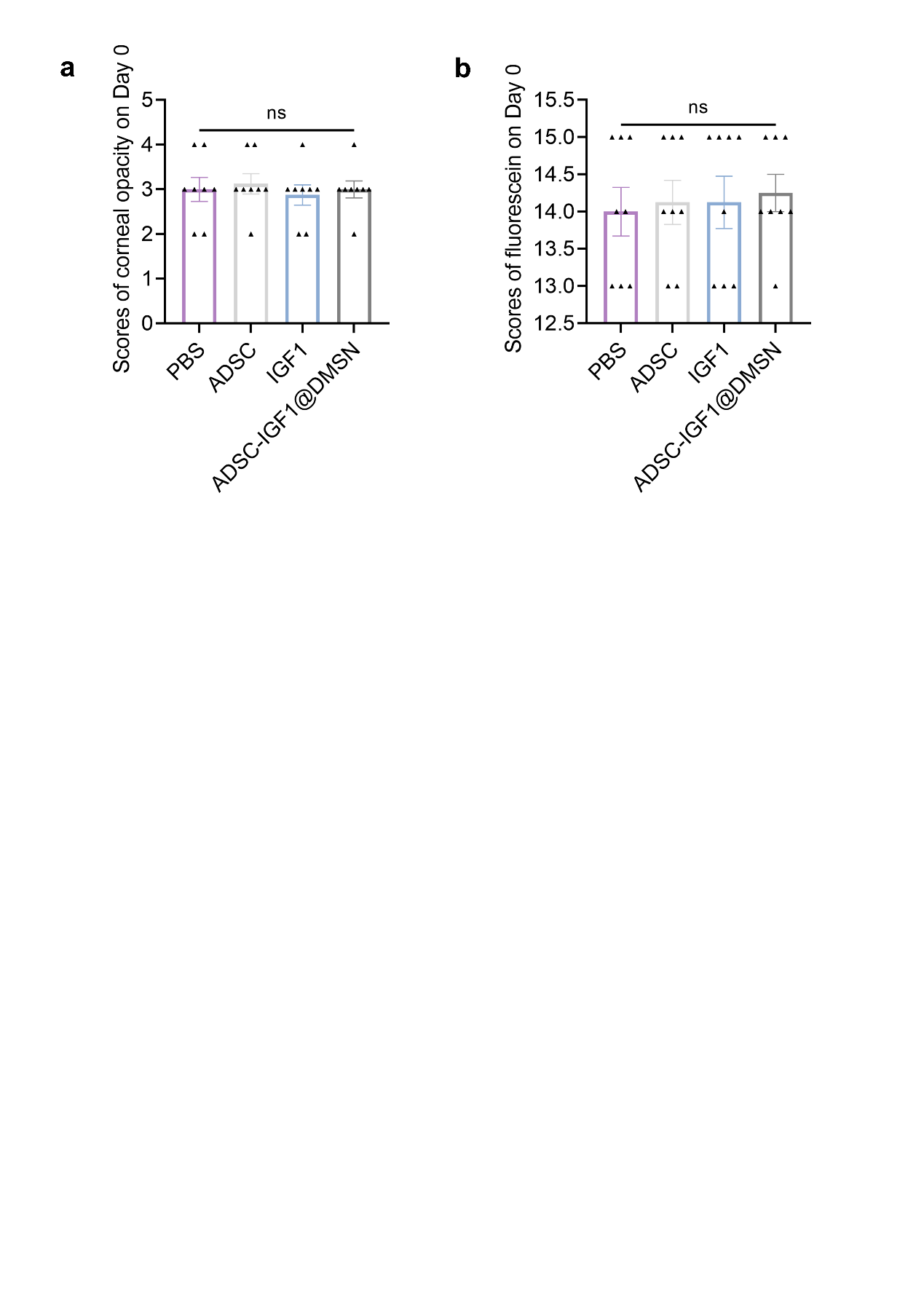


**Fig. S14 a** Quantification of corneal transparency scores at Day 0 post chemical injury. **b** Quantifications of fluorescein staining-based epithelial defect scores at Day 0 post chemical injury. One-way ANOVA was performed for comparison among the groups. n = 8 (a, b).


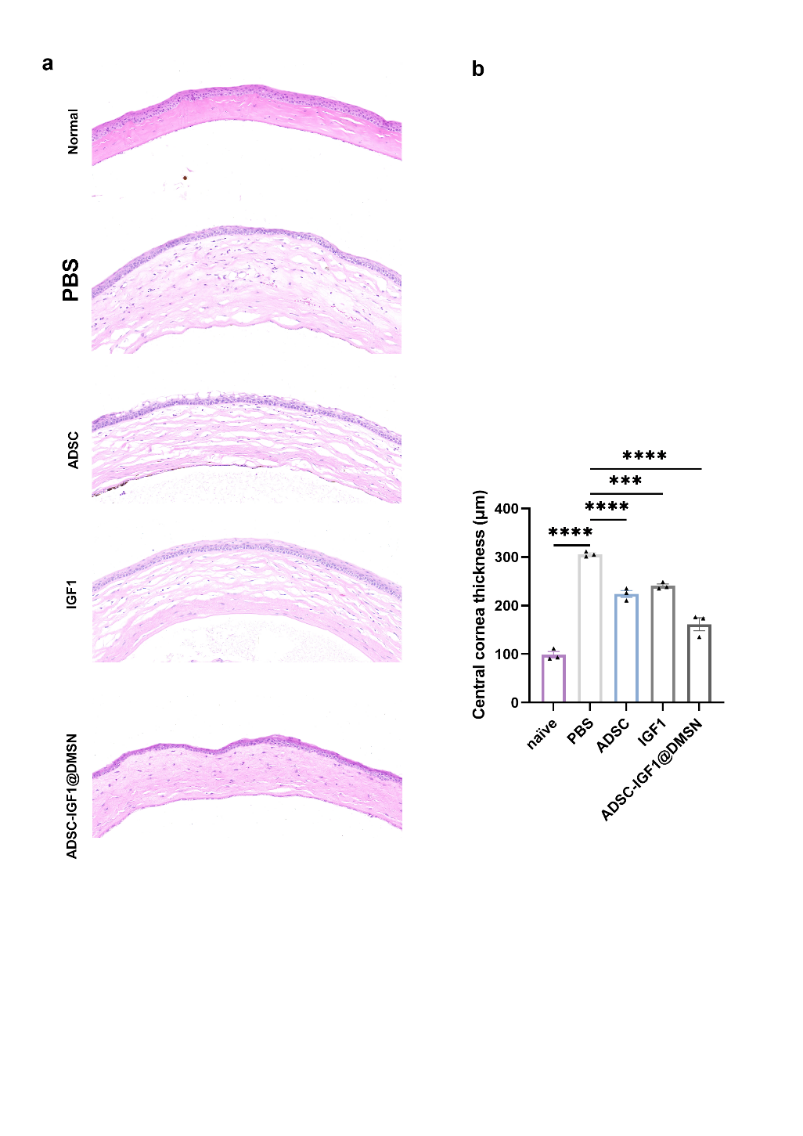


**Fig. S15 a** Corneal tissue morphology after treatment in each group. **b** Central corneal thickness in mice after treatment in each group. One-way ANOVA was performed for comparison among the groups. n=3.


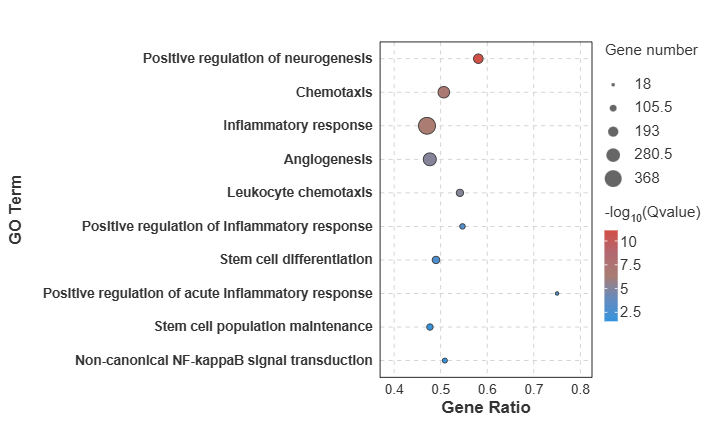


**Fig. S16** GO analysis results for each group of mice corneas.

**
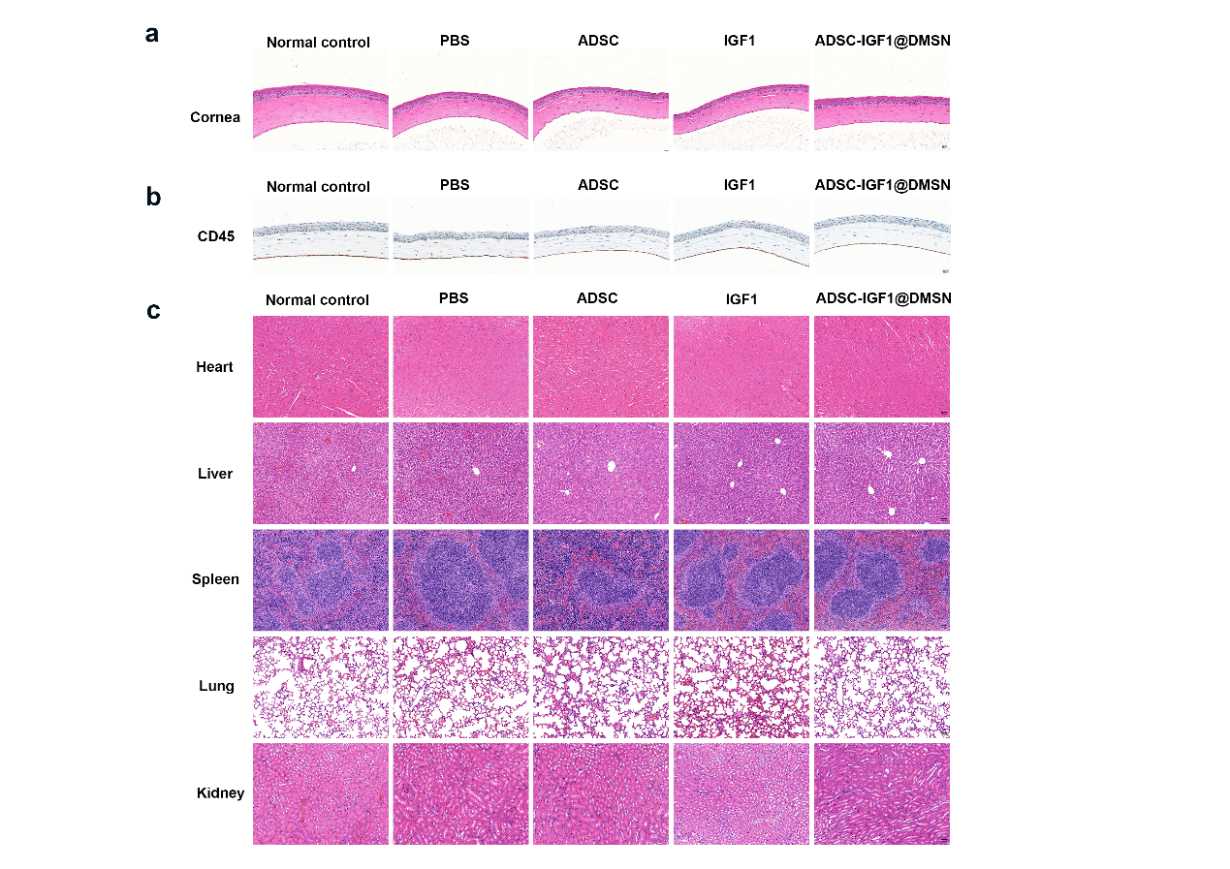
**

**Fig. S17** Non-toxicity testing on the eyeball and major organs of each group. **a** Hematoxylin‑eosin staining of corneal tissues in mice from each group after treatment. **b** Immunohistochemical staining of CD45 in the corneas of mice from each group. **c** Hematoxylin‑eosin staining of major organs (heart, liver, spleen, lung and kidney) in mice from each group**.**

**
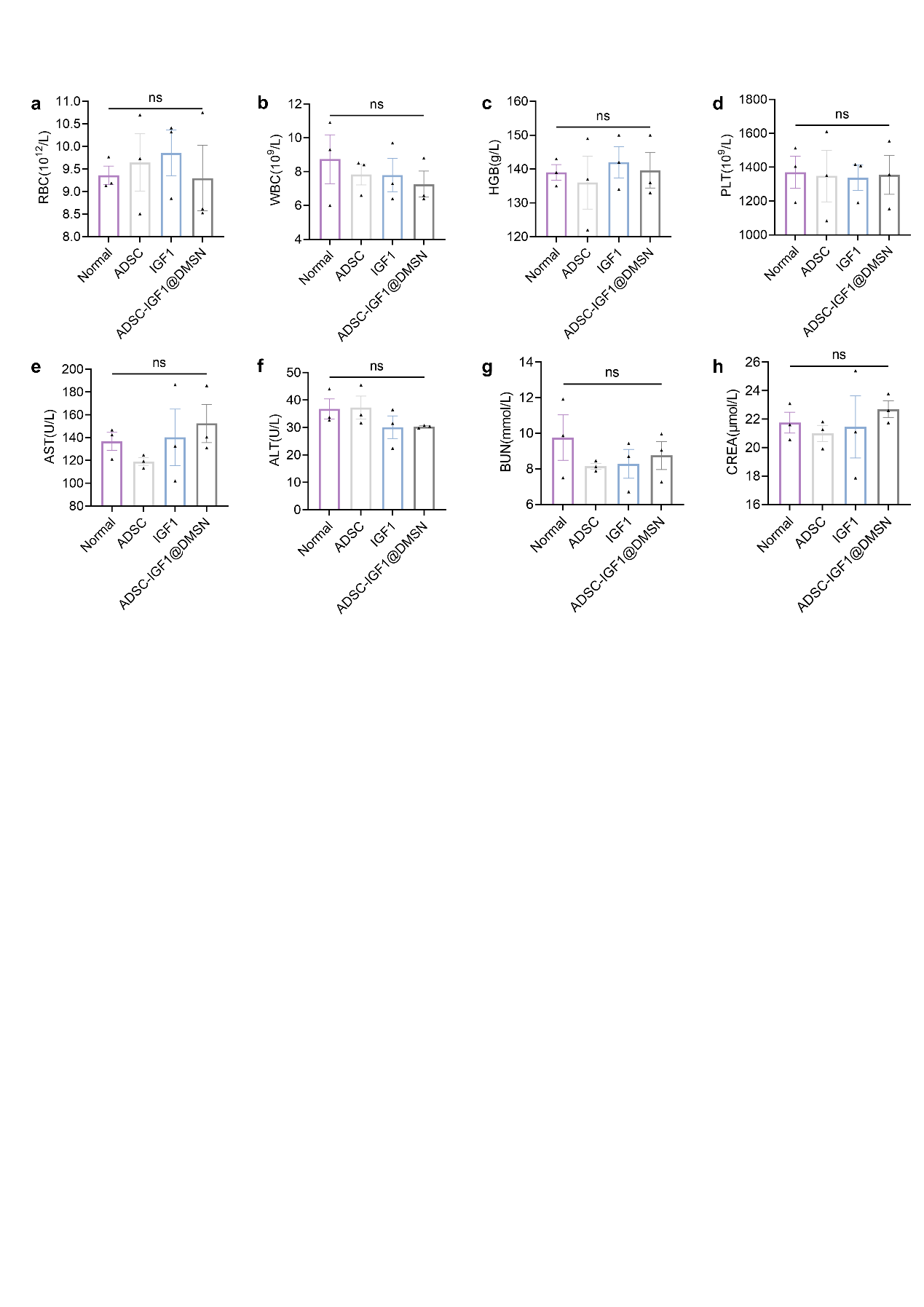
Fig. S18** The routine blood tests and biochemical indicators RBC, WBC, HGB, PLT, ALT, AST, BUN, and CREA of each group. One-way ANOVA was performed for comparison among the groups. n=3.

**Supplementary References**

1. M.C. Scott, C.H. Park, M. Dietrich, X. Wu, J.M. Gimble et al., Isolation of murine adipose-derived stromal/stem cells for adipogenic and osteogenic differentiation or flow cytometry-based analysis. Methods Mol. Biol. **2783**, 93-107 (2024). <https://doi.org/10.1007/978-1-0716-3762-3_6>
2. Y.Q. Chen, Y.C. Shao, R.L. Wei, Pioglitazone alleviates lacrimal gland impairments induced by high-fat diet by suppressing M1 polarization. J. Lipid. Res. **65**, 100606 (2024). <https://doi.org/10.1016/j.jlr.2024.100606>
